# Prior evolution in stochastic versus constant temperatures affects RNA virus evolvability at a thermal extreme

**DOI:** 10.1002/ece3.6287

**Published:** 2020-04-29

**Authors:** Andrea Gloria‐Soria, Sandra Y. Mendiola, Valerie J. Morley, Barry W. Alto, Paul E. Turner

**Affiliations:** ^1^ Department of Ecology and Evolutionary Biology Yale University New Haven CT USA; ^2^ Florida Medical Entomology Laboratory University of Florida Vero Beach FL USA; ^3^ Program in Microbiology Yale School of Medicine New Haven CT USA; ^4^ Present address: Department of Environmental Sciences, Center for Vector Biology and Zoonotic Diseases The Connecticut Agricultural Experiment Station New Haven CT USA; ^5^ Present address: Department of Biology Emory University Atlanta GA 30322 USA; ^6^ Present address: Department of Biology Pennsylvania State University University Park PA 16802 USA

**Keywords:** adaptation, experimental evolution, historical contingency, molecular evolution

## Abstract

It is unclear how historical adaptation versus maladaptation in a prior environment affects population evolvability in a novel habitat. Prior work showed that vesicular stomatitis virus (VSV) populations evolved at constant 37°C improved in cellular infection at both 29°C and 37°C; in contrast, those evolved under random changing temperatures between 29°C and 37°C failed to improve. Here, we tested whether prior evolution affected the rate of adaptation at the thermal‐niche edge: 40°C. After 40 virus generations in the new environment, we observed that populations historically evolved at random temperatures showed greater adaptability. Deep sequencing revealed that most of the newly evolved mutations were de novo. Also, two novel evolved mutations in the VSV glycoprotein and replicase genes tended to co‐occur in the populations previously evolved at constant 37°C, whereas this parallelism was not seen in populations with prior random temperature evolution. These results suggest that prior adaptation under constant versus random temperatures constrained the mutation landscape that could improve fitness in the novel 40°C environment, perhaps owing to differing epistatic effects of new mutations entering genetic architectures that earlier diverged. We concluded that RNA viruses maladapted to their previous environment could “leapfrog” over counterparts of higher fitness, to achieve faster adaptability in a novel environment.

## Graphical abstract

It is unclear how historical adaptation versus maladaptation in a prior environment affects population evolvability in a novel habitat. We tested whether prior evolution of vesicular stomatitis virus (VSV) populations at either at constant temperature or under random changing temperatures affected the rate of adaptation at the thermal‐niche edge. Our results suggest that prior adaptation under constant versus random temperatures constrained the mutation landscape that could improve fitness in the novel environment and conclude that RNA viruses maladapted to their previous environment could “leapfrog” over counterparts of higher fitness, to achieve faster adaptability in a novel environment.

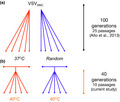

## 1 INTRODUCTION

It is generally assumed that all biological populations experience changes in their environments, at least occasionally. The frequency of environmental turnover can affect which phenotypic variants within a population are favored by natural selection, thus dictating whether the population is relatively homogeneous versus heterogeneous (polymorphic) for genetic variation underlying these phenotypes. Frequent environmental turnover may present differing targets for selection, whereby the relative fitness of certain phenotypes are favored in some environments, but the relative performances of these variants are disfavored in other environments (Buckling, Kassen, Bell, & Rainey, 2000; Kassen, 2002). In this way, an interaction between the tempo of environmental change and the mutations underlying fitness traits may cause a population to harbor greater versus lesser standing genetic variation. Stochastic environmental changes may be particularly challenging for evolving populations, because mismatches between variants and the selective environments can cause genetic variation to accumulate and yet prove insufficient to fuel adaptation (i.e., inability for one or more variants to experience sustained positive selection across varying environments causes adaptation to “stall out,” and increases vulnerability to extinction) (Bürger and Lynch, 1995; Donaldson‐Matasci, Lachmann, & Bergstrom, 2008; Lande & Shannon, 1996; Botero, Weissing, Wright, & Rubenstein, 2015). Thus, stochastic (unpredictable) environments can cause genetic variance to increase in a population over time, without mean fitness improvement (maladaptation). In contrast, selection in a constant environment should cause relatively lower standing genetic variation to be present in an adapting population. Beyond the expected difference in standing variation, the fixation of alleles in a population successfully adapting to its environment should cause its genetic architecture to diverge from a population that is maladapted. By definition, the underlying genetic basis of phenotypic traits that improve fitness (i.e., adaptations) constitutes genetic architecture; therefore, populations that share recent common ancestry but are adapted versus maladapted to their environments should necessarily diverge in genetic architecture, as well as differ in standing variation.

Despite these expected differences, it is unclear whether previous adaptation versus maladaptation in a selective environment should be consequential for evolvability in a novel habitat where all populations are equally unfit. One possibility is that prior successful adaptation molds genetic architecture, such that the population experiences a relatively greater likelihood of conditionally beneficial mutations in the new environment. That is, previously adapted versus maladapted populations can undergo the same spontaneous mutation rate in the novel environment, but the well‐adapted population could more easily evolve via beneficial mutations that modify existing phenotypic traits to achieve more rapid fitness improvement (Bjorklund, 1996; Crespi, 2000; Kirkpatrick & Barton, 1997). This idea seems especially plausible if the novel environment is constant and an extension of the prior habitat (e.g., selection at a novel elevated temperature that is close to the thermal condition of the prior environment). On the other hand, if greater standing genetic variation increases the likelihood that a population will harbor conditionally beneficial alleles in the novel environment, the prior‐maladapted population may experience a relative advantage in evolvability. Importantly, this idea assumes that accumulation of mutations in the prior environment is proportional to the probability that this variation will be useful for fitness in the novel condition. Although some experimental evolution studies have tested the role of historical contingency in further evolution (e.g., Kryazhimskiy, Rice, Jenison, & Desai, 2014; Meyer et al., 2012; Travisano, Mongold, Bennett, & Lenski, 1995; Zachary, Borland, & Lenski, 2008), the evolutionary fate of adapted versus maladapted populations in a novel environment has rarely been tested.

Vesicular stomatitis virus (VSV) is a zoonotic *Vesiculovirus* of veterinary importance in the family *Rhabdoviridae*, which infects domesticated cattle, horses, and swine, and whose definitive natural host reservoir remains unclear (Rozo‐Lopez, Drolet, & Londoño‐Renteria, 2018). The virus is transmitted between hosts via insect vectors, such as mosquitoes, sand flies, black flies, and biting midges, as well as through direct contact (Lyles & Rupprecht, 2006; Rozo‐Lopez et al., 2018). In addition, VSV provides a biological model for studying generalities of RNA virus evolution (Elena et al., 2001; Holland et al., 1991; Morley, Sistrom, Usme‐Ciro, 2016; Remold, Rambaut, & Turner, 2008; Turner & Elena, 2000; Turner, Morales, Alto, & Remold, 2010; Williams et al., 2016). VSV has an ~11kb negative‐sense ssRNA genome, encoding 5 proteins: the nucleocapsid (N) protein that tightly encapsidates the genomic RNA, phosphoprotein (P), and large (L) protein which make up the polymerase, glycoprotein (G) involved in cell‐surface binding and infection initiation via membrane fusion, and matrix (M) protein important for virion formation and inhibition of host antiviral gene expression (Rose & Whitt, 2001). In laboratory tissue culture, replication of wild‐type VSV on baby hamster kidney (BHK) cells is roughly equal at temperatures ranging from 28°C to 37°C, with virus populations achieving equivalent sizes in these environments (Alto & Turner, 2010).

We used VSV and tissue culture cells as a model to compare and contrast phenotypic and molecular evolution of RNA viruses, when evolved in constant versus fluctuating temperature environments for host‐cell infection (Alto, Wasik, Morales, & Turner, 2013). A single ancestral genotype (clone) of VSV was used to found five replicate lineages in a high temperature treatment that imposed constant selection at 37°C and in a random temperature treatment where the selective environment stochastically changed each passage day, in the interval ranging between 29°C and 37°C. The experiment comprised 25 passages total (i.e., 100 generations of VSV evolution; Alto et al., 2013). Results showed that virus populations passaged at 37°C improved in fitness relative to their ancestor, in both the selective environment and via correlated improvement at the lower edge of the thermal niche, 29°C. In contrast, viruses evolved under stochastic temperatures failed to increase in fitness relative to their ancestor at either temperature extremes, 29°C and 37°C (Alto et al., 2013). For these reasons, here we define the constant 37°C ‐evolved virus lineages as adapted to their selective environment, whereas the random temperature‐selected lineages of viruses are maladapted to their prior environment.

Here, we used the two sets of viruses generated by Alto et al. (2013) to test whether prior adaptation versus maladaptation was consequential for the rate of fitness improvement in a novel temperature for host‐cell infection: 40°C. We confirmed that the virus lineages previously passaged under stochastic temperatures harbored relatively greater genetic variation, but that both sets of populations were equally of low fitness in the novel environment. We then created a gene pool (mixture) of variants drawn from populations evolved at constant 37°C and similarly for viruses that previously experienced a random temperature environment. These two gene pools were each used to found five replicate lineages that were passaged for an additional 10 days (40 generations) of virus evolution at 40°C. We determined whether historical contingency (prior adaptation vs. maladaptation) led to differing rates of evolvability in the evolutionary time allowed by the current experiment. In addition, we conducted whole‐genome sequencing of evolved viruses to infer which mutations may be responsible for fitness improvement at 40°C and to discern whether populations showed identical (parallel) versus unique mutational solutions to the common selective problem: improved host‐cell infection at 40°C.

## 2 METHODS

### 2.1 Strains and culture conditions

Baby hamster kidney (BHK) cells were obtained from the laboratory of Esteban Domingo (University of Madrid) and were used as hosts for virus infection. Cells were grown in 6‐well tissue culture plates under Dulbecco's modified Eagle's minimum essential medium (DMEM) with 10% fetal bovine serum and 1% penicillin and streptomycin. Cells were incubated at 37°C, 95% relative humidity, and 5% CO_2_ to achieve confluent monolayers of ~1 × 10^6^ cells/cm^2^. Viruses were originally derived from the Mudd Summer strain of VSV Indiana serotype.

### 2.2 Prior experimental evolution

**FIGURE 1:**
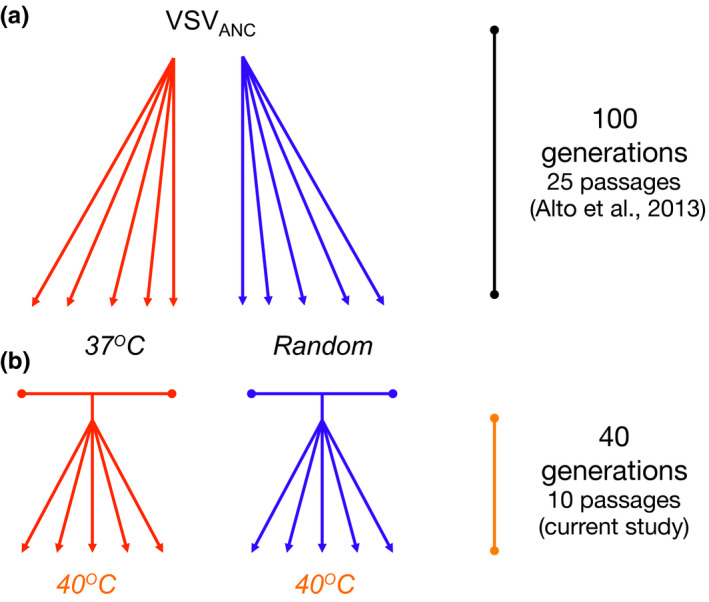
Evolutionary history of the constant (37°C) and random lineages used in this study. (a) Alto et al. (2013) experimental evolution that yielded the constant and random individual lineages that were combined in the current study to generate two ancestral gene pools. (b) Experimental evolution at 40°C of five VSV lineages founded by each ancestral gene pool

All viruses in the current study came from a prior evolution experiment (Alto et al., 2013). Briefly, an ancestral clone of VSV was used to found five replicate populations (lineages) in each of two treatments (Figure 1a). The high temperature treatment imposed constant selection at 37°C. The random treatment imposed a randomly chosen temperature on each passage day, within the interval ranging between 29°C and 37°C (i.e., exposure to 1 of 9 possible temperatures each day with median = 33.2°C; see Figure 1 of Alto et al., 2013). To initiate each treatment population, a cell monolayer was infected with ancestral VSV at a multiplicity of infection (MOI) of 0.001 viruses per cell. After one hour incubation at 37°C, the infected monolayer was incubated for an additional 23 hr according to the treatment regime; the common 37°C adsorption step avoided potential confounding effects of temperature‐dependent differences in virus attachment. After 24 hr, each population was diluted 10^5^‐fold to begin the subsequent passage. This process was repeated for 25 passages total (i.e., ~100 generations of VSV evolution; Miralles, Moya, & Elena, 2000). At each passage, aliquots of supernatant containing the virus progeny were sampled from each lineage and stored at −80°C for future analysis.

### 2.3 Current experimental evolution—selection in novel 40°C environment

In the current study, two ancestral “gene pools” were created, using viruses from the Alto et al., (2013) study (Figure 1b). One ancestral pool contained a mixture of equimolar samples from the five VSV lineages that experienced prior evolution at 37°C treatment; hereafter, this gene pool is referred to as previously selected under Constant temperature. The other ancestral pool was created identically but using the five VSV lineages that experienced random temperature selection. The current experiment used each ancestral pool to initiate five replicate populations (10 populations total; Figure 1b) that were allowed to infect a cell monolayer at MOI = 0.01 plaque‐forming units (PFU) per cell (this MOI was chosen to avoid selection favoring defective‐interfering particles, characteristic of high MOI infections with VSV; Horodyski, Nichol, Spindler, & Holland, 1983). After initial 45‐min incubation at 37°C, the infected monolayers were incubated at 40°C for 23 hr. The next day, each population was diluted 10^4^‐fold to begin the following passage by transferring an aliquot to a fresh BHK monolayer at MOI = 0.01. This process was repeated for a total of 10 passages. At the end of the experiment, aliquots of the supernatant of each virus population were sampled and stored at −80°C for later analysis. We note that mixing samples from the five evolutionary lineages in the 37°C treatment, and similarly combining those from lineages evolved under Random temperatures, ensures that a broad spectrum of mutations arising in the prior treatment conditions are present together in the same ancestral pool in the current study. An alternative design is to preserve treatment‐lineage independence by creating five separate ancestors per treatment group to study adaptation at 40°C. One important difference is that preserving lineage independence should increase the probability that low‐frequency variants in the population are represented in the founding population, potentially affecting adaptability in the new 40°C environment. This possible evolvability effect of pooling lineage samples versus preserving lineage independence is the subject of our future work.

### 2.4 Absolute fitness

Absolute fitness is defined as the observed mean capacity (titer reported as log10 PFU per mL) for a test virus to produce infectious virus particles when grown on cells at MOI = 0.01, where direct virus–virus interactions within coinfected cells are minimized (Wasik, Muñoz‐Rojas, Okamoto, Miller‐Jensen, & Turner, 2016). Absolute fitness was measured by allowing a test virus to infect BHK cells under the above‐described culture conditions, followed by plaque assays of serially diluted aliquots to estimate titer. Aliquots grown on BHK cells were plated under DMEM with 10% heat‐inactivated fetal bovine serum and solidified with 1% low‐melt agarose and incubated for 24 hr at 37°C. After incubation, agarose was removed and cells were stained with crystal violet to visualize plaques. Each plaque was assumed to have originated from a single infecting virus.

### 2.5 RNA isolation, cDNA synthesis, and genome amplification

Genomic viral RNA was extracted from 22 test viruses: 10 lineages from the Alto et al. (2013) study that were used to establish the two ancestral pools in the current experiment (i.e., each endpoint 37°C and random lineage; Figure 1a); two ancestral pools (constant, random); and 10 endpoint (passage 10) VSV lineages in the current study. RNA isolation was performed via QIAamp Viral RNA Mini Kit (Qiagen) and then subjected to cDNA synthesis using *SuperScript II* Reverse Transcriptase (Life Technologies) and random hexamer primers. The majority of the VSV genome was amplified via polymerase chain reaction (PCR) as 8 overlapping fragments ranging between 1 and 2.5kb in length, using primers listed in Appendix S1.

### 2.6 Genome sequencing and bioinformatics

For Sanger sequencing of evolved populations, the resulting PCR fragments were purified using a mixture of 1ul of Exonuclease I (*Exo1*; NEB), 1ul Alkaline phosphatase (NEB), and 1ul of Alkaline phosphatase buffer (NEB) and incubated at 37°C for 20 min followed by 80°C for an additional 20 min. Sequencing was performed via dye termination (Sanger) at the Yale University DNA Analysis Facility at Science Hill using primers listed in Appendix S1. Sequence processing and genome assembly were performed in Geneious 11.1.4 (http://www.geneious.com), and polymorphic sites were confirmed by visual inspection of chromatograms. The resulting sequence represented the population consensus genome. Variable sites within a sample were identified as secondary peaks. However, minority variants were likely missed by this approach, since only high frequency variants were detectable (Remold et al., 2008). Consensus sequences of evolved populations were aligned with each other and with the consensus sequences from the ancestral 37°C and random populations from Alto et al., (2013).

For library preparation and deep sequencing of ancestral populations, Illumina‐sequencing libraries were constructed from the 10 lineages from Alto et al., (2013) that were used to generate the two ancestral pools. For these samples, each step from reverse transcription through sequencing was completed in duplicate, resulting in two technical replicates of each sample. Amplified genome fragments were purified using the QIAquick PCR Purification Kit (Qiagen), and the eight fragments from each virus population were combined to create an equimolar mixture. Libraries were prepared from the pooled viral amplicons using the Nextera XT Kit (Illumina Inc.) and the Nextera Index Kit (Illumina Inc.). Samples were sequenced via paired‐end, 75bp‐read Illumina HiSeq 2000 (Illumina Inc.) at the Yale Center for Genome Analysis. Quality control of reads was conducted using FastQC (Andrews, 2010) with a minimum quality score of 20 and a minimum length of 30bp.

Ion Torrent‐sequencing libraries were constructed from the two ancestral pools (constant, random) and samples from passage 10, for each of the evolved lineages (*N* = 12). Amplified genomes were purified using SPRI beads (Rohland & Reich, 2012) and sheared to an average size of 400bp using a Covaris E210 fragmentation (Covaris). Viral fragments belonging to the same sample were then combined in equimolar concentrations. Barcoded libraries were prepared from each sample pool with the Ion Plus Fragment Library Kit (Life Technologies). Samples were sequenced at the Yale University DNA Analysis Facility at Science Hill, using an Ion PGM^TM^ System (Life Technologies).

### 2.7 Genome assembly and alignment

#### 2.7.1 Illumina sequencing

Illumina sequences were mapped to the publicly available VSV genome (GI:9627229) using BWA v0.7.12 (Li & Durbin, 2009) with default parameters. Consensus sequences and minority‐variant tables were generated using the QUASR pipeline (Watson et al., 2013) with a minimum variant frequency cutoff of 0.01. Only polymorphisms detected in both technical sequencing replicates of each sample were retained to reduce false‐positive results arising during the sequencing process.

#### 2.7.2 Ion Torrent sequencing

Quality of the Ion Torrent runs was inspected using FastQC v.0.11.5 (Andrews, 2010). Reads were quality (‐t 27 –l 30) and size‐filtered (30‐ 250bp) using the FASTX‐Toolkit v.0.0.14 (http://hannonlab.cshl.edu/fastx_toolkit/ *)* and then mapped to the VSV reference genome (GI:9627229) using Bowtie2 v.2.2.9 (Langmead, Trapnell, Pop, & Salzberg, 2009). Average genome coverage was 2123.7X ± 1131.3 (49.2X‐4061.9X), with population B from the random treatment failing to yield reads. Consensus sequences and pileup files were generated with SAMtools v.1.5 (Li and Durbin, 2009; Li et al., 2009) and VCFtools v.0.1.14 (Danecek et al., 2011,). Variable sites were identified using VarScan2 v.2.4.3 (Koboldt et al., 2012); only variants present at frequencies above 1% (0.01) were considered and indels were excluded. Variants at frequencies above 90% were also excluded, since they referred to 107 sites confirmed to have been fixed between the VSV reference genome (GI:9627229) and the ancestral populations from Alto et al., (2013).

Note that while we use two different NGS technologies differing in their manufacturers’ reported error rates (Illumina: 0.001 vs. Ion Torrent: 0.01), Ion Torrent substitution error rates (excluding indels) are an order of magnitude lower (Bragg, Stone, Butler, Hugenholtz, & Tyson, 2013; Huang, Hung, & Wang, 2019) and thus similar to that of Illumina technology. Thus, a minority‐variant filter of <0.01 is appropriate and in agreement with cutoffs suggested by previous reports studying viral quasi‐species (Van den Hoecke, Verhelst, Vuylsteke, & Saelens, 2015).

### 2.8 Statistical analysis

A linear mixed model was used to assess the fixed effect of evolutionary history (historical contingency) and random effect of lineage on viral fitness at the final passage of the experimental evolution at 40°C (PROC MIXED, SAS Institute 2004). One‐way ANOVA and Welch two‐sample *t* test were used for other comparisons (R v. 3.4.0, R Core Team, 2017; Prism 8 v. 8.0.2, GraphPad Software Inc.).

## 3 RESULTS

### 3.1 Differing genetic variation in ancestral gene pools

Sanger sequencing data in the previous study (Alto et al., 2013) suggested that greater average genetic variation was harbored in the five populations evolved in the random temperature treatment, relative to the five lineages evolved at 37°C. These sets of populations were used to create the two ancestral gene pools in the current study (Figure 1).

Next‐generation sequencing (NGS) offers a more powerful approach than Sanger sequencing, to identify rare alleles present at low frequencies in a population. In the current study, we obtained NGS data for the five populations evolved previously at 37°C and for those evolved under random temperatures, to determine whether they differed in average genetic diversity. Consensus sequences derived from NGS confirmed that there are more variable sites across the VSV genomes of lineages in the random treatment (*N* = 13), compared with those that experienced the constant 37°C treatment (*N* = 6) (Files S1, S2). Although the number of minor allele frequency variant sites (MAFV; >1%) was not significantly different between the treatments (t = −0.87959, *df* = 15.328, *p* = .3927; File S3), the frequency spectrum of the MAFVs in the random lineages was shifted toward higher frequencies compared with those from the 37°C treatment, with an average MAFV_Rnd_ frequency of 0.04 and a median of 0.05, compared with an average MAFV_37C_ frequency of 0.02 and a median of 0.03 (t = −3.0237, *df* = 664.88, *p* = .0025; Appendix S2). We concluded that the lineages evolved previously under random temperatures contained greater average allelic diversity than those evolved at strictly 37°C.

We then sought to confirm that the sampling of the sets of evolved populations to create the two ancestral gene pools (Figure 1b) was successful in maintaining the observed difference in mean genetic variation described above. To do so, we generated consensus sequences and conducted variant analysis on NGS data for each of the two ancestral pools created for the current study. Results confirmed that the diversity difference remained after the sets of lineages were combined to create the ancestral pools; the constant ancestral pool showed 71 variable sites, whereas the random ancestral pool presented 100 variable sites (Appendix S3 and S4). We concluded that the random ancestral pool was more genetically variable than the constant ancestral pool.

### 3.2 Equivalent fitness of ancestral pools in the novel 40°C environment

**FIGURE 2:**
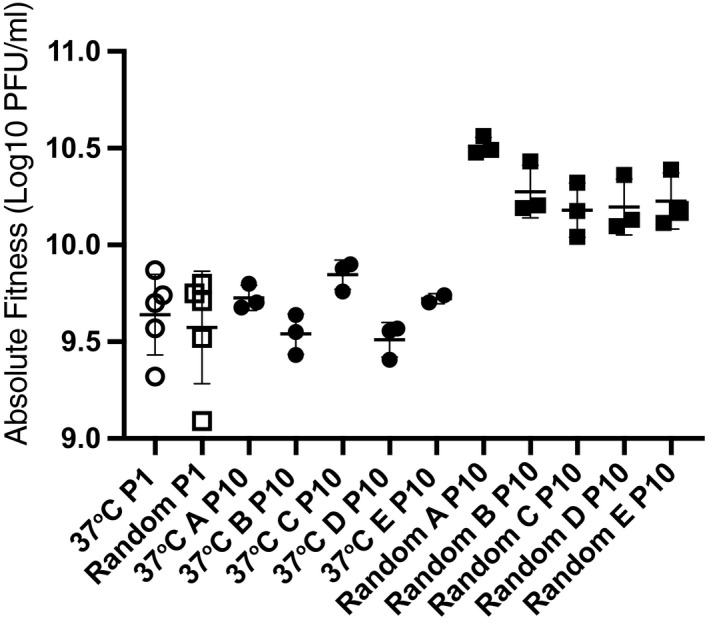
Evolved fitness of VSV lineages after 10 passages at 40°C on BHK cells. Fitness measured via plaque assay as viral growth after 24 hr at 40°C starting with a multiplicity of infection (MOI) of 0.01 particles/cell. Circles are the 37°C lineages and squares the random lineages. Each symbol indicates the mean fitness of a single virus lineage with the 95% CI shown. Fitness of the ancestral strains under the same conditions is indicated by outlined symbols, evolved populations by filled symbols. The random treatment group evolved higher grand mean fitness than the 37°C group (t = −4.8036, *df* = 4.4, *p* = .0068)

Prior to initiation of the current experimental evolution, we used replicated (*n* = 5) fitness assays to test whether the ancestral pools (constant, random) differed in growth performance in the 40°C environment intended to impose virus selection. Results showed that the constant ancestral pool had a mean absolute fitness of 9.64 ± 0.21 s.d.m. log10 PFU/ml, and the ancestral random pool had a mean absolute fitness of 9.57 ± 0.29 s.d.m. log10 PFU/ml; outlined symbols in Figure 2. Comparison of these data revealed that the two ancestral pools did not statistically differ in mean fitness (t = 0.3345, *df* = 7.9, *p* = .74). We concluded that neither ancestral mixture was advantaged in fitness performance in the 40°C selective environment, prior to initiation of the current experimental evolution.

### 3.3 Faster adaptation at 40°C for viruses previously evolved under random temperatures

We hypothesized that prior selection at constant 37°C temperature versus at random temperatures ranging between 29°C and 37°C would impact further ability for RNA viruses to adapt to constant elevated temperature of 40°C (i.e., effect of historical contingency). To do so, we used each ancestral gene pool to create five new lineages that underwent 10 passages (40 virus generations) of selection at 40°C (Figure 1b).

After 10 experimental passages, we used replicated (*n* = 3) fitness assays to measure the average growth performance at 40°C of each endpoint population in the two treatment groups (constant, random). Results (Figure 2) yielded mean log10 absolute fitness values for each constant population (37A: 9.73 ± 0.06 s.d.m.; 37B: 9.54 ± 0.104 s.d.m.; 37C: 9.85 ± 0.07 s.d.m.; 37D: 9.51 ± 0.09 s.d.m.; 37E: 9.72 ± 0.03 s.d.m. log10 PFU/ml). We then compared the dataset for each population to the mean fitness of its founding ancestral gene pool (9.64 ± 0.21 s.d.m. log10 PFU/ml). This statistical analysis showed that none of the 5 populations evolved increased fitness at 40°C relative to the common ancestor (File S4).

Similarly, we obtained mean log10 absolute fitness values (Figure 2) for each random population (RndA: 10.51 ± 0.05 s.d.m.; RndB: 10.27 ± 0.135 s.d.m.; RndC: 10.180 ± 0.14 s.d.m.; RndD: 10.19 ± 0.14 s.d.m.; RndE: 10.23 ± 0.14 s.d.m. log10 PFU/ml). We then compared the dataset for each population to the mean fitness of its founding ancestral gene pool (9.57 ± 0.29 s.d.m. log10 PFU/ml). This statistical analysis showed that all five of these populations evolved increased fitness at 40°C relative to their common ancestor (File S4).

To test the main hypothesis that historical contingency should cause viruses evolved under constant 37°C temperature versus random temperatures to generally differ in adaptability at 40°C, we compared the grand mean fitness improvement for the two sets of evolved populations. Results (Figure 2) showed that the grand mean log10 fitness of the random populations was numerically greater than that of the constant lineages: LS means ± SE: 10.28 ± 0.06 versus 9.67 ± 0.06 PFU/ml. Furthermore, a statistical analysis showed that these two values significantly differed (linear mixed model: F_1,8_ = 47.21, *p* = .0001; t = −4.8036, *df* = 4.4, *p* = .0068). We concluded from the data and analysis that the prior selection under random temperatures led to an adaptive advantage, on average, in fitness gained at 40°C, relative to viruses evolved under prior constant 37°C selection.

### 3.4 Historical selection affects molecular evolution of populations at 40°C

**FIGURE 3:**
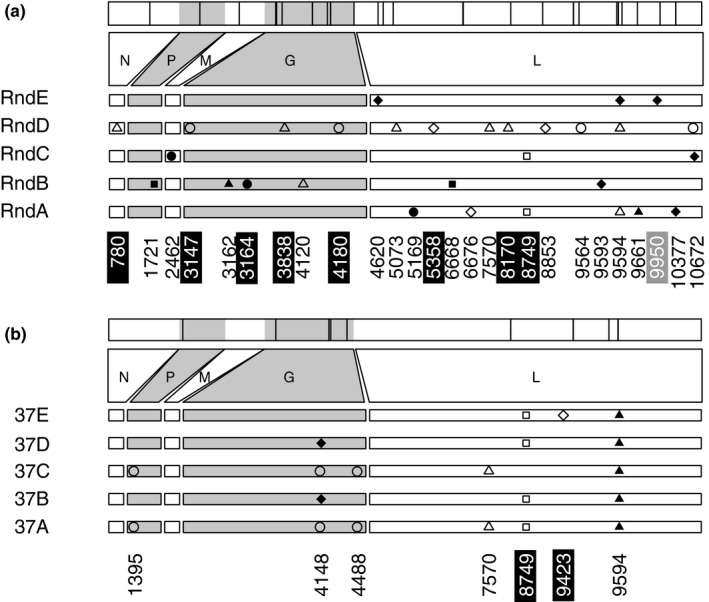
Novel alleles observed in the 10 evolved lineages, relative to the VSV reference genome (GI:9627229). (a) Random pool and (b) 37°C pool. The vertical lines on the top panel are the relative position of the observed mutations in the VSV genome. Numbers on the bottom panel indicate the position of the mutations. Symbols indicate the nucleotide of the derived allele: filled square (T), filled circle (A), filled triangle (G), filled diamond (C), circle outline (M), triangle outline (R), square outline (S), and diamond outline (Y). Highlighted in black are derived alleles detected in the ancestral pool at >1% frequency; in grey are derived alleles not detected in the pool but present at low frequency (>1%) in any of the original populations from Alto et al. (2013) that gave rise to the corresponding pool. VSV genes are shown: nucleoprotein (N), phosphoprotein (P), matrix protein (M), glycoprotein (G), and large protein (L). Alleles known to have fixed between the reference genome and the ancestral lineages from Alto et al. (2013) are not shown

We obtained NGS data (Figure 3) for each of the endpoint populations in the current study, to identify variable sites associated with each treatment group, and to evaluate whether identical (parallel) mutations fixed within and between the two sets of treatment populations.

We observed that consensus sequences of the terminal (passage 10; Figure 3) populations showed different amounts of variation across the two treatment groups. Variant analysis on the NGS data confirmed more variable sites (frequency > 1%) (broader mutational spectrum) in the random lineages (*N* = 123), relative to the 37°C lineages (*N* = 96), although the difference was not significant (t = 1.88, *df* = 6.05, *p* = .1082; Appendix S5 and S6). Interestingly, of the 33 mutations depicted in Figure 3, only 11 were detectable (frequency > 1%) in the respective ancestral gene pools, or detected when inspecting the individual NGS data for the five original lineages that were used to create each of the ancestral gene pools (Figure 1 and Figure 3, File S3, Appendix S3 and S4). These results suggest that the majority of mutations relevant for adaptation to the 40°C environment appeared de novo via spontaneous mutation in the current study, after the populations had entered the new selective environment.

In addition, we observed differing patterns of parallel molecular evolution in our study (Figure 3a and 3b). In particular, greater parallelism was observed in the populations that experienced prior selection at 37°C (Figure 3b). All five of these lineages showed an identical fixed amino acid substitution in the VSV large (L) protein gene (A9594G: Tyr to Cys). Four out of five of these populations also showed an identical fixed or polymorphic change in the VSV glycoprotein (G) gene (A4148C) that does not change the amino acid sequence. In contrast, none of the populations previously evolved under random temperatures showed this allele change in G, and three of these lineages showed evidence of the same change or a different one in the allele position in L (Figure 3a). Otherwise, the random A‐E populations showed no detectable evidence for parallel molecular substitutions.

## 4 DISCUSSION

A fundamental goal of evolutionary biology is to improve its predictive power as a science, such as accurately predicting genotype–phenotype associations, and the future adaptive trajectories of evolving lineages (Graves & Weinreich, 2017; Lassig, Mustonen, & Walczak, 2017). These aims are ambitious, because there are many ways that the effect of a beneficial allele on individual fitness can change over time, making it difficult to predict its long‐term evolutionary fate. For example, the biotic and abiotic environments in which the allele resides may change over time, presenting new challenges that may alter whether allele effects on gene expression, pleiotropy, and epistasis remain beneficial for individual fitness. Moreover, the fitness effects of a beneficial allele can change when additional mutations at other loci cause additions/deletions in the genome, and its effects can be altered if fitness depends on the relative abundance of other genotypes in the population (frequency dependence). Essentially, these myriad variables indicate that the fitness benefit experienced by an allele or genotype can change across generations and environments (e.g., evolutionary landscapes, Remold, 2012), making the long‐term success of an allele and the persistence of its lineage difficult to predict.

Experimental evolution studies offer useful “proving grounds” to test how biological populations adapt to new environmental challenges, and to gauge whether theory accurately predicts how current performance relates to future evolution (Garland & Rose, 2009). Here, we showed that virus population adaptation and maladaptation in a selective environment were consequential for fitness improvements in a novel environment. Specifically, RNA virus populations evolved in a constant high temperature (37°C) were disadvantaged in evolvability at the thermal niche edge (40°C), compared to their counterparts with a history of evolution in stochastic (random) temperatures. Furthermore, we found that virus populations previously evolved in stochastic temperatures contained greater genetic variation coupled with their greater adaptability than virus populations evolved in constant high temperature (37°C). Our empirical studies support the notion that RNA viruses maladapted to a previous environment (i.e., random populations) may have a fitness advantage in novel environments, in part, through access to conditionally beneficial alleles which may influence their adaptive potential (McBride & Turner, 2008; evolutionary revolutions, Lenormand, Roze, & Rousset, 2009). Theory predicts and empirical observations support the notion that fluctuating thermal environments should enhance tolerance at thermal extremes (Duncan, Fellous, Quillery, & Kaltz, 2011; Levins, 1968; Lynch & Gabriel, 1987). Thus, historical contingency may facilitate preadaptation and the emergence of organisms (pathogens) in novel environments (Arnold, Jackson, Waterfield, & Mansfield, 2007; Ketola et al., 2013; Lee & Gelembiuk, 2008). On the one hand, the repeatability of this outcome suggests it might be helpful for the overall goal of refining our power to make evolutionary predictions: Lineages that perform relatively worse in their current environment can still be potentiated to “leapfrog” in evolvability over counterpart lineages historically evolved near the new selective target. On the other hand, it is unknown whether the result would be repeatable if the new environment was slightly different, such as adaptation to cellular infection at 38°C or 39°C or adaptation to another type of stressful environment (pH, salinity). For example, populations of *Drosophila melanogaster* selected for increased desiccation resistance showed correlated responses to resistance to other stressors including heat shock and starvation (Hoffman & Parsons, 1989a, b). Other experiments on viral adaptation to novel hosts have tested whether host‐selection regimes result in positive or negative correlated performance in either the original or unselected (novel) hosts, and multiple outcomes have been observed (Cooper & Scott, 2001; Greene et al., 2005; Novella, Hershey, Escarmis, Domingo, & Holland, 1999; Turner & Elena, 2000; Weaver, Brault, Kang, & Holland, 1999; Zárate & Novella, 2004). This dichotomy is precisely the reason why historical contingency and evolvability studies are intriguing and vital areas for future work. If underlying patterns truly exist, it may be plausible (albeit dismaying) to conceive that advancements may only come from increased sheer effort: use more attempts to examine effects of historical contingency on evolvability in empirical studies across various biological systems, and observe whether patterns emerge. Notably, differences in the current study design might have unknown effects on the observed outcome. We created gene pools for the 37°C and random temperature evolved populations and sub‐sampled from each pool to establish the replicate populations challenged to evolve at 40°C; this design emphasized the effect of relatively low (37°C evolved) versus high (random evolved) genetic variation on 40°C adaptation, when initial diversity of replicate populations is sampled from the same source pool. Alternatively, each population from the prior study could have served as an independent replicate in the current experiment, emphasizing the role of low versus high diversity on adaptation, when founding pools differ by a greater extent. Likewise, different outcomes may have resulted from imposed alternative randomly changing temperature regimes each experimental passage, or if the period between temperature switches had been modified. A possible future study could examine these alternative designs, to test whether it changes the observed outcome.

Our study capitalized on using the set of RNA viruses in Alto et al., (2013) as an empirical model to test the general scenario of populations well‐adapted versus maladapted in their current environments, and whether this differing prior history affected rate of fitness improvement under a new challenge. Environmental fluctuations (e.g., random temperatures) can either cause maladaptation or select for adaptive generalism, which may fortuitously increase ability to invade novel environments (Ketola et al., 2013); whereas a static environment (e.g., constant 37°C) tends to select for specialists (Kassen, 2002). Furthermore, the genetic architectures of populations evolved under these scenarios also should differ. For example, a population selected in environments A and B should have a different genetic signature than a population selected in environment A or B alone, attributable to mutations with fitness trade‐offs that are unlikely to fix in a population selected in both environments. Our data are consistent with the idea that populations selected in stochastically fluctuating (random) environments are advantaged at niche limits (Kassen, 2002; Levins, 1968). Also, altered thermal tolerance limits have been observed in *E. coli* (“stepping stone” and “sliding niche” hypotheses, Mongold, Bennett, & Lenski, 1996; Mongold, Bennett, & Lenski, 1999) and *Drosophila* (Huey, Partridge, & Fowler, 1991; Watson & Hoffmann, 1996) following selection in higher or fluctuating temperatures. Although the current study was not intended to test a specific circumstance in the evolution of natural virus populations, our results could help inform how virus adaptation might proceed in roughly analogous situations.

Foremost, it is clear that predictions of RNA virus emergence pose important and difficult challenges in biomedicine and public health, and we believe that the results of our study are both relevant and useful for refining emergence predictions. In particular, certain bat species are known to serve as animal reservoirs of RNA viruses that emerge as zoonotic disease agents in humans and other mammals (Calisher, Childs, Field, Holmes, & Schountz, 2006). Bats in the family *Pteropodidae* (commonly known as “flying foxes”) can harbor henipaviruses, such as Hendra virus and Nipah virus, with high case‐fatality rates when these viruses spillover into hosts such as horses and humans. It is unclear whether particular physiological and behavioral traits can best explain why some bat species tend to serve as RNA‐virus reservoirs. The fluctuating body temperatures of bats offer one key possibility: the body temperatures of bats can be very low when daily roosting and during extended hibernation, whereas these temperatures can be greatly elevated when bats are actively flying and foraging for food (Calisher et al., 2006). Because viremias in apparently healthy bats are observed to be unaffected by these temperature extremes, the immunobiology of bats has received attention to explain why these mammals can harbor diverse communities of viruses without succumbing to illness (e.g., Xie et al., 2018; Zhang et al., 2013). Regardless of the explanatory mechanism, our study suggests a related phenomenon that may merit attention. Our observed results support the concept that RNA viruses with greater access to genetic variation enhanced evolvability compared to RNA viruses with less genetic variation when encountering novel environments distinct from the prior habitat. The apparent persistence of RNA virus populations and communities within individual bats might create diverse variant pools that fail to reach high titers (i.e., viremias within bats are constant but do not reach the high loads expected when a virus overwhelms the host immune system). The relatively diverse viruses evolved in stochastic (random) temperatures in the Alto et al., (2013) study could be viewed as analogous to those within a bat reservoir. By further analogy, the observed evolvability advantage of these viruses in a constant new environment (40°C) might be similar to the success of viruses that spillover from bats into novel hosts, such as humans and horses, with constant and higher body temperatures than those experienced in the bat reservoirs. For this reason, it might be useful to test how the genetic variance of important emerging RNA viruses is affected when cultured in the laboratory under constant versus fluctuating temperatures, and whether this history impacts performance and evolution at novel temperatures seen in endpoint (emergence) hosts.

Our observed findings that some previously selected RNA virus populations (random populations) have improved performance at the thermal niche limits may be applicable to another real‐life example relevant to public health. Cold‐adapted genotypes of Influenza A virus may have a selective advantage in terms of increased survival outside of the preferred niche, potentially contributing to additional cases and flu epidemics (Hatta et al., 2007; Hayashi, Wills, Bussey, & Takimoto, 2015; Massin, Werf, & Naffakh, 2001). Virulent H5N1 influenza variants have a replicative advantage and are more tolerant of cooler temperatures in the upper respiratory tract of mammals attributable to an amino acid change in the PB2 protein.

The genetic differences that separated the evolved lineages from their ancestral gene pools suggested that genetic architectures and epistasis may have played key roles. The virus populations historically evolved in random temperatures achieved relatively higher fitness in the novel 40°C environment in the time allowed, but these lineages showed few similarities to one another in terms of genetic changes that could account for their highly similar phenotypic improvements. This result clearly indicates that relatively more genetic “solutions” existed for these populations to meet the adaptation challenge, relative to the obvious parallelism observed among the 37°C ‐evolved populations. Moreover, the latter 37°C ‐evolved populations did not manage to improve in fitness in the new environment, on average, despite the strong selection to evolve similar—and fewer—solutions to the selection problem. So, prior adaptive success among the 37°C ‐evolved populations constrained mutational solutions in the novel temperature environment. Altogether, these data suggest that the prior experiment by Alto et al., (2013) somehow molded the populations drawn from the two treatments to experience two very different outcomes in the current study.

## CONFLICT OF INTERESTS

The authors declare no competing interests.

## AUTHOR'S CONTRIBUTIONS

**Andrea Gloria‐Soria:** Data curation (lead); formal analysis (lead); investigation (lead); visualization (lead); writing – original draft (lead); writing – review & editing (equal). **Sandra Y. Mendiola:** Data curation (equal); investigation (equal); writing – review & editing (equal). **Valerie J. Morley:** Data curation (equal); formal analysis (equal); methodology (equal); writing – review & editing (equal). **Barry W. Alto:** Formal analysis (equal); methodology (equal); validation (equal); writing – review & editing (equal). **Paul E. Turner:** Conceptualization (lead); funding acquisition (lead); investigation (equal); methodology (equal); project administration (lead); resources (equal); supervision (equal); writing – original draft (equal); writing – review & editing (equal).

## Supporting information

File S1

File S2

File S3

File S4

Appendix S1‐S6

## Data Availability

Tables and consensus sequence are available in the Appendix or as supplementary files S1–S4. Raw Illumina and Ion Torrent sequence reads are available through Dryad https://doi.org/10.5061/dryad.qf0bs33.

## References

[ece36287-bib-0001] Alto, B. W. , & Turner, P. E. (2010). Consequences of host adaptation for performance of vesicular stomatitis virus in novel thermal environments. Evolutionary Ecology, 24(2), 299–315. 10.1007/s10682-009-9307-3

[ece36287-bib-0002] Alto, B. W. , Wasik, B. R. , Morales, N. M. , & Turner, P. E. (2013). Stochastic temperatures impede RNA virus adaptation. Evolution, 67(4), 969–979. 10.1111/evo.12034 23550749

[ece36287-bib-0003] Andrews, S. , (2010). FastQC: A quality control tool for high throughput sequence data.

[ece36287-bib-0004] Arnold, D. , Jackson, W. , Waterfield, N. R. , & Mansfield, J. W. (2007). Evolution of microbial virulence: The benefits of stress. Trends in Genetics, 23, 293–300. 10.1016/j.tig.2007.03.017 17434232

[ece36287-bib-0005] Bjorklund, M. (1996). The importance of evolutionary constraints in ecological time scales. Evolutionary Ecology, 10, 423–431. 10.1007/BF01237727

[ece36287-bib-0006] Botero, C. A. , Weissing, F. J. , Wright, J. , & Rubenstein, D. R. (2015). Evolutionary tipping points in the capacity to adapt to environmental change. Proceedings of the National Academy of Sciences of the United States of America, 112, 184–189. 10.1073/pnas.1408589111 25422451 PMC4291647

[ece36287-bib-0007] Bragg, L. M. , Stone, G. , Butler, M. K. , Hugenholtz, P. , & Tyson, G. W. (2013). Shining a light on dark sequencing: Characterising errors in Ion Torrent PGM data. PLoS Computational Biology, 9(4), e1003031 10.1371/journal.pcbi.1003031 23592973 PMC3623719

[ece36287-bib-0008] Buckling, A. , Kassen, R. , Bell, G. , & Rainey, P. B. (2000). Disturbance and diversity in experimental microcosms. Nature, 408, 961–964. 10.1038/35050080 11140680

[ece36287-bib-0066] Bürger R. , Lynch M. (1995) Evolution and extinction in a changing environment: A quantitative‐genetic analysis. Evolution, 49(1), 151–163. 28593664 10.1111/j.1558-5646.1995.tb05967.x

[ece36287-bib-0009] Calisher, C. H. , Childs, J. E. , Field, H. E. , Holmes, K. V. , & Schountz, T. (2006). Bats: Important reservoir hosts of emerging viruses. Clinical Microbiology Reviews, 19(3), 531–545. 10.1128/cmr.00017-06 16847084 PMC1539106

[ece36287-bib-0010] Cooper, L. A. , & Scott, T. W. (2001). Differential evolution of eastern equine encephalitis virus populations in response to host cell type. Genetics, 157, 1403–1412. 11290699 10.1093/genetics/157.4.1403 PMC1461603

[ece36287-bib-0011] Crespi, B. J. (2000). The evolution of maladaptation. Heredity, 84, 623–629. 10.1046/j.1365-2540.2000.00746.x 10886377

[ece36287-bib-0012] Danecek, P. , Auton, A. , Abecasis, G. , Albers, C. A. , Banks, E. , DePristo, M. A. , … Durbin, R. (2011). The variant call format and VCFtools. Bioinformatics, 27(15), 2156–2158. 10.1093/bioinformatics/btr330 21653522 PMC3137218

[ece36287-bib-0013] Donaldson‐Matasci, M. C. , Lachmann, M. , & Bergstrom, C. T. (2008). Phenotypic diversity as an adaptation to environmental uncertainty. Evolutionary Ecology Research, 10, 493–515.

[ece36287-bib-0014] Duncan, A. B. , Fellous, S. , Quillery, E. , & Kaltz, O. (2011). Adaptation of *Paramecium caudatum* to variable conditions of temperature stress. Research in Microbiology, 162, 939–944. 10.1016/j.resmic.2011.04.012 21575715

[ece36287-bib-0015] Garland, T. Jr , & Rose, M. R. (2009). Experimental evolution: Concepts, methods and applications of selection experiments. Berkeley, CA: University of California Press.

[ece36287-bib-0016] Graves, C. J. , & Weinreich, D. M. (2017). Variability in fitness effects can preclude selection of the fittest. Annual Review of Ecology, Evolution, and Systematics, 48(1), 399–417. 10.1146/annurev-ecolsys-110316-022722 PMC6768565 31572069

[ece36287-bib-0017] Greene, I. P. , Wang, E. , Deardorff, E. R. , Milleron, R. , Domingo, E. , & Weaver, S. C. (2005). Effect of alternating passage on adaptation of Sindbis virus to vertebrate and invertebrate cells. Journal of Virology, 79, 14253–14260. 10.1128/JVI.79.22.14253-14260.2005 16254360 PMC1280187

[ece36287-bib-0018] Hatta, M. , Hatta, Y. , Kim, J. H. , Watanabe, S. , Shinya, K. , Nguyen, T. , … Kawaoka, Y. (2007). Growth of H5N1 influenza A viruses in the upper respiratory tracts of mice. PLoS Path, 3, 1374–1379. 10.1371/journal.ppat.0030133 PMC2000968 17922570

[ece36287-bib-0019] Hayashi, T. , Wills, S. , Bussey, K. A. , & Takimoto, T. (2015). Identification of Influenza A virus PB2 residue involved in enhanced polymerase activity and virus growth in mammalian cells at low temperatures. Journal of Virology, 89(15), 8042–8049. 26018156 10.1128/JVI.00901-15 PMC4505657

[ece36287-bib-0020] Hoffmann, A. A. , & Parsons, P. A. (1989a). An integrated approach to environmental stress tolerance and life‐history variation: Desiccation tolerance in *Drosophila* . Biological Journal of the Linnaean Society, 37, 117–136. 10.1111/j.1095-8312.1989.tb02098.x

[ece36287-bib-0021] Hoffmann, A. A. , & Parsons, P. A. (1989b). Selection for increased desiccation resistance in *Drosophila melanogaster*: Additive genetic control and correlated responses for other stresses. Genetics, 122, 837–845. 2503423 10.1093/genetics/122.4.837 PMC1203758

[ece36287-bib-0067] Holland J. J. , de la Torre J. C. , Clarke D. K. , Duarte E. (1991). Quantitation of relative fitness and great adaptability of clonal populations of RNA viruses. Journal of Virology, 65(6), 2960–2967. 2033662 10.1128/jvi.65.6.2960-2967.1991 PMC240937

[ece36287-bib-0022] Horodyski, F. M. , Nichol, S. T. , Spindler, K. R. , & Holland, J. J. (1983). Properties of DI particle resistant mutants of vesicular stomatitis virus isolated from persistent infections and from undiluted passages. Cell, 33(3), 801–810. 10.1016/0092-8674(83)90022-3 6307527

[ece36287-bib-0023] Huang, S. W. , Hung, S. J. , & Wang, J. R. (2019). Application of deep sequencing methods for inferring viral population diversity. Journal of Virological Methods, 266, 95–102. 10.1016/j.jviromet.2019.01.013 30690049

[ece36287-bib-0024] Huey, R. B. , Partridge, L. , & Fowler, K. (1991). Thermal sensitivity of *Drosophila melanogaster* responds rapidly to laboratory natural selection. Evolution, 45, 751–756. 10.1111/j.1558-5646.1991.tb04343.x 28568829

[ece36287-bib-0025] Kassen, R. (2002). The experimental evolution of specialists, generalists, and the maintenance of diversity. Journal of Evolutionary Biology, 15, 173–190. 1046/j.1420‐9101.2002.00377.x

[ece36287-bib-0026] Ketola, T. , Mikonranta, L. , Zhang, J. , Saarinen, K. , Ӧrmälä, A.‐M. , Friman, V.‐P. , … Laakso, J. (2013). Fluctuating temperature leads to evolution of thermal generalism and preadaptation to novel environments. Evolution, 67(10), 2936–2944. 10.1111/evo.12148 24094344

[ece36287-bib-0027] Kirkpatrick, M. , & Barton, N. H. (1997). Evolution of a species' range. American Naturalist, 150, 1–23. 10.1086/286054 18811273

[ece36287-bib-0028] Koboldt, D. C. , Zhang, Q. , Larson, D. E. , Shen, D. , McLellan, M. D. , Lin, L. , … Wilson, R. K. (2012). VarScan 2: Somatic mutation and copy number alteration discovery in cancer by exome sequencing. Genome Research, 22(3), 568–576. 10.1101/gr.129684.111 22300766 PMC3290792

[ece36287-bib-0029] Kryazhimskiy, S. , Rice, D. P. , Jenison, E. R. , & Desai, M. M. (2014). Global epistasis makes adaptation predictable despite sequence‐level stochasticity. Science, 344, 1519–1522. 10.1126/science.1250939 24970088 PMC4314286

[ece36287-bib-0030] Lande, R. , & Shannon, S. (1996). The role of genetic variation in adaptation and population persistence in a changing environment. Evolution, 50(1), 434–437. 10.1111/j.1558-5646.1996.tb04504.x 28568879

[ece36287-bib-0031] Langmead, B. , Trapnell, C. , Pop, M. , & Salzberg, S. L. (2009). Ultrafast and memory‐efficient alignment of short DNA sequences to the human genome. Genome Biology, 10, R25. 19261174 10.1186/gb-2009-10-3-r25 PMC2690996

[ece36287-bib-0032] Lassig, M. , Mustonen, V. , & Walczak, A. M. (2017). Predicting Evolution. Nature Ecology and Evolution, 1(3), 77. 28812721 10.1038/s41559-017-0077

[ece36287-bib-0033] Lee, C. E. , & Gelembiuk, G. W. (2008). Evolutionary origins of invasive populations. Evolutionary Applications, 1, 427–448. 10.1111/j.1752-4571.2008.00039.x 25567726 PMC3352381

[ece36287-bib-0034] Lenormand, T. , Roze, D. , & Rousset, F. (2009). Stochasticity in evolution. Trends in Ecology and Evolution, 24(3), 157–165. 19178980 10.1016/j.tree.2008.09.014

[ece36287-bib-0035] Levins, R. (1968). Evolution in changing environments: Some theoretical explorations. Princeton, NJ: Princeton University Press.

[ece36287-bib-0036] Li, H. , & Durbin, R. (2009). Fast and accurate short read alignment with Burrows‐Wheeler transform. Bioinformatics, 25, 1754–1760. 10.1093/bioinformatics/btp324 19451168 PMC2705234

[ece36287-bib-0037] Li, H. , Handsaker, B. , Wysoker, A. , Fennell, T. , Ruan, J. , Homer, N. , … Durbin, R. (2009). The sequence alignment/map format and SAMtools. Bioinformatics, 25(16), 2078–2079. 10.1093/bioinformatics/btp352 19505943 PMC2723002

[ece36287-bib-0038] Lyles, D. S. , & Rupprecht, C. E. (2006). Rhabdoviridae In Knipe D. M. et al. Fields Virology (pp. 1363–1408). Philadelphia, PA: Lippincott, Williams and Wilkins.

[ece36287-bib-0039] Lynch, M. , & Gabriel, W. (1987). Environmental tolerance. American Naturalist, 129, 283–303. 10.1086/432558 16224689

[ece36287-bib-0040] Massin, P. , van der Werf, S. , & Naffakh, N. (2001). Residue 627 of PB2 is a determinant of cold sensitivity in RNA replication of avian influenza viruses. Journal of Virology, 75(11), 5398–5404. 10.1128/jvi.75.11.5398-5404.2001 11333924 PMC114948

[ece36287-bib-0041] McBride, R. C. , & Turner, P. E. (2008). Genetic robustness and adaptability of viruses. Microbe, 3, 409–415. 10.1128/microbe.3.409.1

[ece36287-bib-0043] Meyer, J. R. , Dobias, D. T. , Weitz, J. S. , Barrick, J. E. , Quick, R. T. , & Lenski, R. E. (2012). Repeatability and contingency in the evolution of a key innovation in phage Lambda. Science, 335, 428–432. 10.1126/science.1214449 22282803 PMC3306806

[ece36287-bib-0044] Miralles, R. , Moya, A. , & Elena, S. F. (2000). Diminishing returns of population size in the rate of RNA virus adaptation. Journal of Virology, 74, 3566–3571. 10.1128/JVI.74.8.3566-3571.2000 10729131 PMC111865

[ece36287-bib-0045] Mongold, J. A. , Bennett, A. F. , & Lenski, R. E. (1996). Evolutionary adaptation to temperature. IV. Adaptation of *Escherichia coli* at a niche boundary. Evolution, 50, 35–43. 28568880 10.1111/j.1558-5646.1996.tb04470.x

[ece36287-bib-0046] Mongold, J. A. , Bennett, A. F. , & Lenski, R. E. (1999). Evolutionary adaptation to temperature. VII. Extension of the upper thermal limit of *Escherichia coli* . Evolution, 53, 386–394. 10.1111/j.1558-5646.1999.tb03774.x 28565406

[ece36287-bib-0047] Morley, V. , Sistrom, M. , Usme‐Ciro, J. A. , Remold, S. K. , & Turner, P. E. (2016). Evolution in spatially mixed host environments increases divergence for evolved fitness and intrapopulation genetic diversity in RNA viruses. Virus Evolution, 2(1), vev022 10.1093/ve/vev022 27774292 PMC4989875

[ece36287-bib-0048] Novella, I. S. , Hershey, C. L. , Escarmis, C. , Domingo, E. , & Holland, J. (1999). Lack of evolutionary stasis during alternating replication of an arbovirus in insect and mammalian cells. Journal of Molecular Biology, 287, 459–465. 10.1006/jmbi.1999.2635 10092452

[ece36287-bib-0049] R Core Team . (2017). R: A language and environment for statistical computing. Vienna, Austria: R Foundation for Statistical Computing Retrieved from https://www.R‐project.org.

[ece36287-bib-0050] Remold, S. (2012). Understanding specialism when the jack of all trades can be the master of all. Proceedings of the Royal Society B, 279, 4861–4869. 10.1098/rspb.2012.1990 23097515 PMC3497242

[ece36287-bib-0051] Remold, S. K. , Rambaut, A. , & Turner, P. A. (2008). Evolutionary genomics of host adaptation in vesicular stomatitis virus. Molecular Biology and Evolution, 25, 1138–1147. 10.1093/molbev/msn059 18353798

[ece36287-bib-0052] Rohland, N. , & Reich, D. (2012). Cost‐effective, high‐throughput DNA sequencing libraries for multiplexed target capture. Genome Research, 22(5), 939–946. 10.1101/gr.128124.111 22267522 PMC3337438

[ece36287-bib-0069] Rose, J. K. & Whitt, M. A. (2001). Rhabdoviridae: The viruses and their replication In Fields B. N. et al. Fields Virology (pp. 1221–1244). Philadelphia, PA: Lippincott, Williams and Wilkins.

[ece36287-bib-0053] Rozo‐Lopez, P. , Drolet, B. S. , & Londoño‐Renteria, B. (2018). Vesicular stomatitis transmission: A comparison of incriminated vectors. Insects, 9, 190. 10.3390/insects9040190 PMC6315612 30544935

[ece36287-bib-0054] Travisano, M. , Mongold, J. A. , Bennett, A. F. , & Lenski, R. E. (1995). Experimental tests of the roles of adaptation, chance, and history in evolution. Science, 267, 87–90. 10.1126/science.7809610 7809610

[ece36287-bib-0055] Turner, P. E. , & Elena, S. F. (2000). Cost of host radiation in an RNA virus. Genetics, 156, 1465–1470. 11102349 10.1093/genetics/156.4.1465 PMC1461356

[ece36287-bib-0068] Turner P. E. , Morales N. M. , Alto B. W. , & Remold S. K. . (2010). Role of evolved host breadth in the initial emergence of an RNA virus. Evolution, 64(11), 3273–3286. 20633045 10.1111/j.1558-5646.2010.01051.x

[ece36287-bib-0056] Van den Hoecke, S. , Verhelst, J. , Vuylsteke, M. , & Saelens, X. (2015). Analysis of the genetic diversity of influenza A viruses using next‐generation DNA sequencing. BMC Genomics, 16(1), 79 10.1186/s12864-015-1284-z 25758772 PMC4342091

[ece36287-bib-0057] Wasik, B. R. , Muñoz‐Rojas, A. R. , Okamoto, K. W. , Miller‐Jensen, K. , & Turner, P. E. (2016). Generalized selection to overcome innate immunity selects for host breadth in an RNA virus. Evolution, 70, 270–281. 10.1111/evo.12845 26882316

[ece36287-bib-0058] Watson, M. J. O. , & Hoffmann, A. A. (1996). Acclimation, cross‐generation effects, and the response to selection for increased cold resistance in *Drosophila* . Evolution, 50, 1182–1192. 10.1111/j.1558-5646.1996.tb02359.x 28565271

[ece36287-bib-0059] Watson, S. J. , Welkers, M. R. A. , Depledge, D. P. , Coulter, E. , Breuer, J. M. , de Jong, M. D. , & Kellam, P. (2013). Viral population analysis and minority‐variant detection using short read next‐generation sequencing. Philosophical Transactions of the Royal Society B: Biological Sciences, 368(1614), 20120205 10.1098/rstb.2012.0205 PMC3678329 23382427

[ece36287-bib-0060] Weaver, S. C. , Brault, A. C. , Kang, W. , & Holland, J. J. (1999). Genetic and fitness changes accompanying adaptation of an arbovirus to vertebrate and invertebrate cells. Journal of Virology, 73, 4316–4326. 10.1128/JVI.73.5.4316-4326.1999 10196330 PMC104213

[ece36287-bib-0061] Williams, E. S. C. P. , Morales, N. M. , Wasik, B. R. , Brusic, V. , Whelan, S. P. J. , & Turner, P. E. (2016). Repeatable population dynamics among vesicular stomatitis virus lineages evolved under high co‐infection. Frontiers in Microbiology, 7, 370 10.3389/fmicb.2016.00370 27065953 PMC4815288

[ece36287-bib-0062] Xie, J. , Li, Y. , Shen, X. , Goh, G. , Zhu, Y. , Cui, J. , … Zhou, P. (2018). Dampened STING‐dependent interferon activation in bats. Cell Host and Microbe, 23(3), 297–301. 10.1016/j.chom.2018.01.006 29478775 PMC7104992

[ece36287-bib-0063] Zachary, D. B. , Borland, C. Z. , & Lenski, R. E. (2008). Historical contingency and the evolution of a key innovation in an experimental population of *Escherichia coli* . Proceedings of the National Academy of Sciences of the United States of America, 105(23), 7899–7906. 18524956 10.1073/pnas.0803151105 PMC2430337

[ece36287-bib-0064] Zárate, S. , & Novella, I. S. (2004). Vesicular stomatitis virus evolution during alternation between persistent infection in insect cells and acute infection in mammalian cells is dominated by the persistence phase. Journal of Virology, 78, 12236–12242. 10.1128/JVI.78.22.12236-12242.2004 15507610 PMC525086

[ece36287-bib-0065] Zhang, G. , Cowled, C. , Shi, Z. , Huang, Z. , Bishop‐Lilly, K. A. , Fang, X. , … Wang, J. (2013). Comparative analysis of bat genomes provides insight into the evolution of flight and immunity. Science, 339(6118), 456–460. 10.1126/science.1230835 23258410 PMC8782153

