# Supplementary material for: Prior evolution in stochastic versus constant temperatures affects RNA virus evolvability at a thermal extreme
Source: Ecol Evol. 2020 Apr 29;10(12):5440–50. doi: 10.1002/ece3.6287 (PMC7319105; doi:10.1002/ece3.6287)
Supplement: Supplementary file 5 — Appendix S1‐S6 [file ECE3-10-5440-s005.docx]

**APPENDIX 1**: Primers used to generate and sequence overlapping fragments of the Vesicular Stomatitis Virus (VSV) genome via polymerase-chain reaction.

| **Fragment** | **F Primer** | **R Primer** | **~ Length (kb)** |
| --- | --- | --- | --- |
| A | 27F | 1547F | 1.5 |
| B | 987F | 2844R | 1.9 |
| C | 3189F | 4968R | 1.8 |
| D | 2502F | 3663R | 1.1 |
| E | 4332F | 6096R | 1.8 |
| F | 5196F | 7251R | 2.1 |
| G | 6957F | 9062R | 2.1 |
| H | 8756F | 11143R | 2.4 |

**APPENDIX 2**: Minor allele frequency variant (MAFV) spectrum of the ancestral populations evolved at constant 37^o^C (red) and Random (blue) temperature regimes; regions where both colors overlap appear in purple. Small panel shows a zoomed section of the main plot.


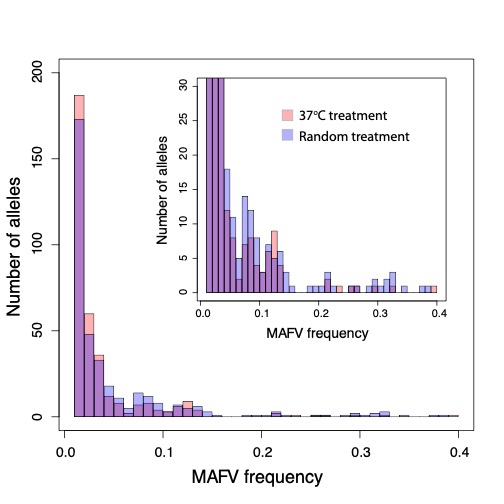


**APPENDIX 3**: 37^o^C ancestral pool variants. CHR: chromosome; POS: position, REF: reference allele; ALT: alternative (variant) allele; VARFREQ: frequency of the variant allele.

CHR POS REF ALT VARFREQ

gi|9627229|ref|NC_001560.1| 222 . A G 1.33%

gi|9627229|ref|NC_001560.1| 968 . A T 4.95%

gi|9627229|ref|NC_001560.1| 1529 . T C 1.22%

gi|9627229|ref|NC_001560.1| 1530 . G A 1.57%

gi|9627229|ref|NC_001560.1| 1538 . A T 1.03%

gi|9627229|ref|NC_001560.1| 1539 . T G 1.04%

gi|9627229|ref|NC_001560.1| 1540 . A T 1.03%

gi|9627229|ref|NC_001560.1| 1542 . T A 1.04%

gi|9627229|ref|NC_001560.1| 1653 . C T 4.97%

gi|9627229|ref|NC_001560.1| 1789 . G A 1.71%

gi|9627229|ref|NC_001560.1| 1877 . C A 1.28%

gi|9627229|ref|NC_001560.1| 1959 . G A 1.56%

gi|9627229|ref|NC_001560.1| 2255 . T G 1.2%

gi|9627229|ref|NC_001560.1| 2584 . G T 1.96%

gi|9627229|ref|NC_001560.1| 3114 . G C 4.09%

gi|9627229|ref|NC_001560.1| 3148 . A G 30.21%

gi|9627229|ref|NC_001560.1| 3164 . C A 1.08%

gi|9627229|ref|NC_001560.1| 3268 . G T 13.72%

gi|9627229|ref|NC_001560.1| 3290 . C A 1.48%

gi|9627229|ref|NC_001560.1| 3444 . C T 2.27%

gi|9627229|ref|NC_001560.1| 3445 . C G 2.15%

gi|9627229|ref|NC_001560.1| 3447 . G A 2.4%

gi|9627229|ref|NC_001560.1| 3451 . T C 2.75%

gi|9627229|ref|NC_001560.1| 3452 . C T 2.64%

gi|9627229|ref|NC_001560.1| 3453 . C T 2.35%

gi|9627229|ref|NC_001560.1| 3456 . C G 2.18%

gi|9627229|ref|NC_001560.1| 3457 . C T 2.15%

gi|9627229|ref|NC_001560.1| 3459 . C G 2.2%

gi|9627229|ref|NC_001560.1| 3461 . A C 2.92%

gi|9627229|ref|NC_001560.1| 3462 . A G 1.02%

gi|9627229|ref|NC_001560.1| 3465 . T C 1.24%

gi|9627229|ref|NC_001560.1| 3466 . G C 2.66%

gi|9627229|ref|NC_001560.1| 3467 . T A 1.98%

gi|9627229|ref|NC_001560.1| 3469 . G C 1.08%

gi|9627229|ref|NC_001560.1| 3471 . T G 1.09%

gi|9627229|ref|NC_001560.1| 3578 . A G 1.31%

gi|9627229|ref|NC_001560.1| 3602 . C T 1.03%

gi|9627229|ref|NC_001560.1| 3681 . A T 36.12%

gi|9627229|ref|NC_001560.1| 4180 . C A 65.97%

gi|9627229|ref|NC_001560.1| 4390 . G C 74.72%

gi|9627229|ref|NC_001560.1| 4870 . T C 1.85%

gi|9627229|ref|NC_001560.1| 5176 . T A 7.45%

gi|9627229|ref|NC_001560.1| 5297 . A C 1.08%

gi|9627229|ref|NC_001560.1| 5345 . C G 2.66%

gi|9627229|ref|NC_001560.1| 5347 . C T 2.94%

gi|9627229|ref|NC_001560.1| 5710 . A C 1.08%

gi|9627229|ref|NC_001560.1| 6080 . A G 3%

gi|9627229|ref|NC_001560.1| 6895 . G A 16.42%

gi|9627229|ref|NC_001560.1| 6937 . T A 5.86%

gi|9627229|ref|NC_001560.1| 7056 . A T 2.21%

gi|9627229|ref|NC_001560.1| 7058 . T G 2.24%

gi|9627229|ref|NC_001560.1| 7061 . G A 1.06%

gi|9627229|ref|NC_001560.1| 7062 . G A 1.22%

gi|9627229|ref|NC_001560.1| 7064 . A T 1.49%

gi|9627229|ref|NC_001560.1| 7065 . A T 1.04%

gi|9627229|ref|NC_001560.1| 7067 . A T 2.76%

gi|9627229|ref|NC_001560.1| 7068 . T C 1.96%

gi|9627229|ref|NC_001560.1| 7072 . G T 3.13%

gi|9627229|ref|NC_001560.1| 7073 . A C 3.1%

gi|9627229|ref|NC_001560.1| 7074 . T C 3.95%

gi|9627229|ref|NC_001560.1| 7075 . T A 3.03%

gi|9627229|ref|NC_001560.1| 7080 . G T 3.04%

gi|9627229|ref|NC_001560.1| 7271 . T A 1.23%

gi|9627229|ref|NC_001560.1| 7292 . C A 1.27%

gi|9627229|ref|NC_001560.1| 8736 . C T 1.33%

gi|9627229|ref|NC_001560.1| 8749 . G C 44.2%

gi|9627229|ref|NC_001560.1| 9082 . T A 2.64%

gi|9627229|ref|NC_001560.1| 9350 . A T 1.5%

gi|9627229|ref|NC_001560.1| 9423 . T C 1.56%

gi|9627229|ref|NC_001560.1| 9588 . C A 1.03%

gi|9627229|ref|NC_001560.1| 11135 . T A 2.56%

**APPENDIX 4**: Random ancestral pool variants. CHR: chromosome; POS: position, REF: reference allele; ALT: alternative (variant) allele; VARFREQ: frequency of the variant allele.

CHR POS REF ALT VARFREQ

gi|9627229|ref|NC_001560.1| 642 . A G 1.2%

gi|9627229|ref|NC_001560.1| 780 . A G 7.51%

gi|9627229|ref|NC_001560.1| 946 . C T 1%

gi|9627229|ref|NC_001560.1| 968 . A T 1.44%

gi|9627229|ref|NC_001560.1| 1020 . T G 1.16%

gi|9627229|ref|NC_001560.1| 1044 . T A 1.2%

gi|9627229|ref|NC_001560.1| 1183 . G T 1.47%

gi|9627229|ref|NC_001560.1| 1186 . G A 1.23%

gi|9627229|ref|NC_001560.1| 1187 . T A 1.31%

gi|9627229|ref|NC_001560.1| 1192 . G A 1.5%

gi|9627229|ref|NC_001560.1| 1195 . T G 1.63%

gi|9627229|ref|NC_001560.1| 1200 . C A 1.95%

gi|9627229|ref|NC_001560.1| 1201 . G T 1.23%

gi|9627229|ref|NC_001560.1| 1206 . G A 1.38%

gi|9627229|ref|NC_001560.1| 1209 . T C 1.29%

gi|9627229|ref|NC_001560.1| 1210 . G T 1.24%

gi|9627229|ref|NC_001560.1| 1213 . G C 1.42%

gi|9627229|ref|NC_001560.1| 1214 . A C 1.42%

gi|9627229|ref|NC_001560.1| 1266 . C A 1.02%

gi|9627229|ref|NC_001560.1| 1594 . G T 20%

gi|9627229|ref|NC_001560.1| 2667 . T C 4.84%

gi|9627229|ref|NC_001560.1| 2915 . G T 7.45%

gi|9627229|ref|NC_001560.1| 3114 . G C 4.1%

gi|9627229|ref|NC_001560.1| 3147 . C A 7.34%

gi|9627229|ref|NC_001560.1| 3148 . A G 3.88%

gi|9627229|ref|NC_001560.1| 3164 . C A 2.01%

gi|9627229|ref|NC_001560.1| 3166 . G A 2.05%

gi|9627229|ref|NC_001560.1| 3249 . C A 5.79%

gi|9627229|ref|NC_001560.1| 3268 . G T 32.25%

gi|9627229|ref|NC_001560.1| 3285 . G T 7.22%

gi|9627229|ref|NC_001560.1| 3377 . C A 3.13%

gi|9627229|ref|NC_001560.1| 3380 . C G 2.8%

gi|9627229|ref|NC_001560.1| 3383 . T C 2.09%

gi|9627229|ref|NC_001560.1| 3384 . C T 2.24%

gi|9627229|ref|NC_001560.1| 3386 . A T 2.91%

gi|9627229|ref|NC_001560.1| 3392 . A C 2.33%

gi|9627229|ref|NC_001560.1| 3393 . G C 2.33%

gi|9627229|ref|NC_001560.1| 3395 . A C 2.72%

gi|9627229|ref|NC_001560.1| 3398 . A G 2.69%

gi|9627229|ref|NC_001560.1| 3399 . T A 2.94%

gi|9627229|ref|NC_001560.1| 3403 . A T 1.47%

gi|9627229|ref|NC_001560.1| 3404 . G T 1.46%

gi|9627229|ref|NC_001560.1| 3407 . A C 1.44%

gi|9627229|ref|NC_001560.1| 3416 . A T 1.52%

gi|9627229|ref|NC_001560.1| 3418 . A C 1.62%

gi|9627229|ref|NC_001560.1| 3420 . A C 1.77%

gi|9627229|ref|NC_001560.1| 3422 . G A 1.82%

gi|9627229|ref|NC_001560.1| 3423 . A G 1.64%

gi|9627229|ref|NC_001560.1| 3425 . A G 1.72%

gi|9627229|ref|NC_001560.1| 3444 . C T 2.21%

gi|9627229|ref|NC_001560.1| 3445 . C G 1.69%

gi|9627229|ref|NC_001560.1| 3447 . G A 1.59%

gi|9627229|ref|NC_001560.1| 3451 . T C 1.77%

gi|9627229|ref|NC_001560.1| 3452 . C T 1.52%

gi|9627229|ref|NC_001560.1| 3453 . C T 1.66%

gi|9627229|ref|NC_001560.1| 3454 . C T 1.11%

gi|9627229|ref|NC_001560.1| 3456 . C A 3.86%

gi|9627229|ref|NC_001560.1| 3457 . C T 3.89%

gi|9627229|ref|NC_001560.1| 3459 . C G 3.58%

gi|9627229|ref|NC_001560.1| 3461 . A C 4.24%

gi|9627229|ref|NC_001560.1| 3462 . A G 1.4%

gi|9627229|ref|NC_001560.1| 3464 . T G 1.08%

gi|9627229|ref|NC_001560.1| 3465 . T C 1.22%

gi|9627229|ref|NC_001560.1| 3466 . G C 4.06%

gi|9627229|ref|NC_001560.1| 3467 . T A 1.81%

gi|9627229|ref|NC_001560.1| 3469 . G C 1.21%

gi|9627229|ref|NC_001560.1| 3471 . T G 1.59%

gi|9627229|ref|NC_001560.1| 3472 . A G 1.06%

gi|9627229|ref|NC_001560.1| 3474 . G C 1.15%

gi|9627229|ref|NC_001560.1| 3477 . A C 1.17%

gi|9627229|ref|NC_001560.1| 3479 . T A 1.22%

gi|9627229|ref|NC_001560.1| 3481 . T A 1.11%

gi|9627229|ref|NC_001560.1| 3484 . C G 1.1%

gi|9627229|ref|NC_001560.1| 3486 . G A 1.33%

gi|9627229|ref|NC_001560.1| 3510 . G C 1.14%

gi|9627229|ref|NC_001560.1| 3512 . G T 1.93%

gi|9627229|ref|NC_001560.1| 3513 . A T 1.29%

gi|9627229|ref|NC_001560.1| 3516 . C G 1.07%

gi|9627229|ref|NC_001560.1| 3648 . G A 1.25%

gi|9627229|ref|NC_001560.1| 3838 . A G 15.69%

gi|9627229|ref|NC_001560.1| 4180 . C A 81.29%

gi|9627229|ref|NC_001560.1| 5335 . G T 4.14%

gi|9627229|ref|NC_001560.1| 5339 . A T 3.32%

gi|9627229|ref|NC_001560.1| 5345 . C G 14.41%

gi|9627229|ref|NC_001560.1| 5347 . C T 13.95%

gi|9627229|ref|NC_001560.1| 5354 . G A 1.36%

gi|9627229|ref|NC_001560.1| 5356 . T A 1.26%

gi|9627229|ref|NC_001560.1| 5358 . C A 1.08%

gi|9627229|ref|NC_001560.1| 5363 . T C 2.54%

gi|9627229|ref|NC_001560.1| 5365 . T G 6.75%

gi|9627229|ref|NC_001560.1| 5366 . A G 2.43%

gi|9627229|ref|NC_001560.1| 5431 . G T 1.23%

gi|9627229|ref|NC_001560.1| 5704 . A G 2.09%

gi|9627229|ref|NC_001560.1| 6240 . C A 1.52%

gi|9627229|ref|NC_001560.1| 6536 . A T 1.13%

gi|9627229|ref|NC_001560.1| 7497 . A C 7.92%

gi|9627229|ref|NC_001560.1| 7902 . G T 3.51%

gi|9627229|ref|NC_001560.1| 8170 . G A 3.55%

gi|9627229|ref|NC_001560.1| 8749 . G C 23.01%

gi|9627229|ref|NC_001560.1| 9082 . T A 7.45%

**APPENDIX 5**: Variants present in each of the evolved 37^o^ C lineages after 10 passages at 40^o^C . CHR: chromosome; POS: position, REF: reference allele; ALT: alternative (variant) allele; VARFREQ: frequency of the variant allele at each lineage.

CHR POS REF ALT VARFREQ

37_10D 37_10C 37_10B 37_10A 37_10E

gi|9627229|ref|NC_001560.1| 468 . T C 0% 0% 0.27% 0.28% 1.15%

gi|9627229|ref|NC_001560.1| 698 . C A 0.26% 0.26% 0% 0% 2.11%

gi|9627229|ref|NC_001560.1| 708 . C T 0.26% 0% 0% 2.05% 0.25%

gi|9627229|ref|NC_001560.1| 966 . G C 6.03% 1.88% 1.44% 0% 0.49%

gi|9627229|ref|NC_001560.1| 1016 . A T 0% 0.26% 0.26% 0% 10.36%

gi|9627229|ref|NC_001560.1| 1054 . G A 0% 0.27% 1.85% 2.14% 0.27%

gi|9627229|ref|NC_001560.1| 1228 . C G 0% 1.34% 0.52% 1.09% 0.58%

gi|9627229|ref|NC_001560.1| 1395 . C A 0.27% 45.75% 0.56% 55.41% 8.99%

gi|9627229|ref|NC_001560.1| 1459 . G A 0% 0% 0% 9.11% 0%

gi|9627229|ref|NC_001560.1| 1525 . G T 0.26% 0% 0% 0% 1.89%

gi|9627229|ref|NC_001560.1| 1800 . A C 0.31% 6.59% 1.81% 0.64% 0.52%

gi|9627229|ref|NC_001560.1| 1802 . C G 0.28% 11.11% 2.62% 0.31% 0.54%

gi|9627229|ref|NC_001560.1| 1803 . C T 0% 7.25% 1.53% 0.27% 0.47%

gi|9627229|ref|NC_001560.1| 1806 . G A 0% 9.31% 2.36% 0.28% 0%

gi|9627229|ref|NC_001560.1| 1900 . A G 0% 1.76% 0.27% 0.27% 0%

gi|9627229|ref|NC_001560.1| 1905 . G A 0.28% 1.89% 2.28% 0% 0%

gi|9627229|ref|NC_001560.1| 1951 . A C 0.26% 0% 2.07% 0% 0.44%

gi|9627229|ref|NC_001560.1| 2516 . C T 0% 0.27% 1.80% 0% 0%

gi|9627229|ref|NC_001560.1| 2533 . A G 2.10% 0.26% 1.04% 1.57% 0.52%

gi|9627229|ref|NC_001560.1| 2630 . T C 0.26% 0% 4.06% 0% 0%

gi|9627229|ref|NC_001560.1| 2943 . A G 1.59% 0.26% 0% 0% 0.26%

gi|9627229|ref|NC_001560.1| 2971 . C T 0.28% 24.72% 0.53% 0% 0.53%

gi|9627229|ref|NC_001560.1| 3098 . A G 0.26% 0.25% 0.26% 0% 16.89%

gi|9627229|ref|NC_001560.1| 3099 . GC G TC 6.62% 3.32% 3.10% 4.82% 3.39%

gi|9627229|ref|NC_001560.1| 3114 . G C 0.52% 1.04% 1.05% 2.65% 3.11%

gi|9627229|ref|NC_001560.1| 3445 . C G 0.29% 0% 1.40% 0.29% 0.85%

gi|9627229|ref|NC_001560.1| 3451 . T C 0.63% 0.32% 1.51% 0.31% 0.94%

gi|9627229|ref|NC_001560.1| 3452 . C T 0.26% 0% 1.04% 0.27% 1.07%

gi|9627229|ref|NC_001560.1| 3453 . C T 0.52% 0% 1.29% 0.26% 0.77%

gi|9627229|ref|NC_001560.1| 3456 . C G 0.26% 0.79% 1.03% 0.26% 0.78%

gi|9627229|ref|NC_001560.1| 3459 . C G 0.79% 0.81% 1.30% 0.26% 0.52%

gi|9627229|ref|NC_001560.1| 3461 . A C 1.04% 0.78% 2.54% 0.52% 1.27%

gi|9627229|ref|NC_001560.1| 3463 . G GCTC A 0.80% 0.82% 1.03% 0.26% 0.78%

gi|9627229|ref|NC_001560.1| 3464 . T A G 0.26% 0.26% 1.28% 0.79% 0.77%

gi|9627229|ref|NC_001560.1| 3466 . G C A 1.09% 1.07% 2.33% 0.53% 1.31%

gi|9627229|ref|NC_001560.1| 3467 . T A 0.27% 0.27% 1.55% 0.52% 0.52%

gi|9627229|ref|NC_001560.1| 3470 . A G 0% 0% 1.81% 0% 0.80%

gi|9627229|ref|NC_001560.1| 3818 . C T 0.76% 2.45% 0.81% 1.12% 0.45%

gi|9627229|ref|NC_001560.1| 4074 . A C 1.14% 1.10% 0.82% 2.17% 1.37%

gi|9627229|ref|NC_001560.1| 4148 . A C 95% 34.55% 99.19% 48.03% 6.08%

gi|9627229|ref|NC_001560.1| 4180 . C A 99.74% 98.20% 99.24% 99.49% 99.23%

gi|9627229|ref|NC_001560.1| 4330 . C A 0.26% 0.26% 0.26% 0% 1.04%

gi|9627229|ref|NC_001560.1| 4390 . G C 97.84% 96.44% 98.39% 95.69% 97.83%

gi|9627229|ref|NC_001560.1| 4395 . T A 1.29% 1.26% 0.38% 2.31% 0.42%

gi|9627229|ref|NC_001560.1| 4413 . C A 0% 0.26% 0% 0% 1.06%

gi|9627229|ref|NC_001560.1| 4439 . A C 0% 1.84% 0% 0% 0%

gi|9627229|ref|NC_001560.1| 4488 . A C 0.27% 36.44% 0.26% 46.70% 9.67%

gi|9627229|ref|NC_001560.1| 4694 . T A 0% 0.28% 0.29% 0.29% 1.14%

gi|9627229|ref|NC_001560.1| 4834 . G A 0% 0.52% 3.66% 0% 0%

gi|9627229|ref|NC_001560.1| 4924 . G A 0.27% 0% 0% 3% 0.55%

gi|9627229|ref|NC_001560.1| 4978 . A T 0% 15.28% 0.27% 0% 0.27%

gi|9627229|ref|NC_001560.1| 5113 . G A 1.31% 0.52% 0% 0% 0.26%

gi|9627229|ref|NC_001560.1| 5345 . C G 0.28% 0.54% 0% 0.80% 2.08%

gi|9627229|ref|NC_001560.1| 5347 . C T 0.47% 1.02% 0.54% 1.40% 2.43%

gi|9627229|ref|NC_001560.1| 5425 . G A 0.52% 0.26% 1.06% 0.26% 15.40%

gi|9627229|ref|NC_001560.1| 5707 . G T 0.26% 2.41% 0% 0% 0.27%

gi|9627229|ref|NC_001560.1| 6286 . A G 0.51% 0.26% 10.53% 0.26% 0%

gi|9627229|ref|NC_001560.1| 6532 . A C 4.27% 0.26% 0.27% 0.26% 0%

gi|9627229|ref|NC_001560.1| 6604 . T G 0.29% 2.25% 0% 0.28% 0%

gi|9627229|ref|NC_001560.1| 6681 . A C 1.39% 0% 0.29% 6.18% 0.27%

gi|9627229|ref|NC_001560.1| 6685 . A G 0% 0% 1.90% 0% 0%

gi|9627229|ref|NC_001560.1| 7105 . C T 0% 0% 0.59% 0% 2.29%

gi|9627229|ref|NC_001560.1| 7177 . T C 0.26% 0% 2.08% 0.26% 0%

gi|9627229|ref|NC_001560.1| 7390 . A C 0.27% 0% 0.26% 3.02% 0%

gi|9627229|ref|NC_001560.1| 7429 . C A 0.31% 0.63% 0.90% 1.75% 1.41%

gi|9627229|ref|NC_001560.1| 7536 . T G 0.53% 0.79% 0.26% 1.85% 1.83%

gi|9627229|ref|NC_001560.1| 7570 . A G 0% 40.83% 0% 53.87% 9.70%

gi|9627229|ref|NC_001560.1| 7698 . C T 0.53% 0.52% 1.88% 0.80% 0.54%

gi|9627229|ref|NC_001560.1| 7701 . T A 0.53% 0.52% 1.33% 0.54% 0.52%

gi|9627229|ref|NC_001560.1| 7708 . G C 0.52% 0.53% 1.31% 0.53% 0.52%

gi|9627229|ref|NC_001560.1| 8143 . C T 0.26% 0.52% 3.48% 0.26% 0.28%

gi|9627229|ref|NC_001560.1| 8371 . C T 0.84% 1.44% 3.69% 1.14% 1.10%

gi|9627229|ref|NC_001560.1| 8624 . G A 0.26% 0% 0.56% 0% 1.16%

gi|9627229|ref|NC_001560.1| 8749 . G C 2.36% 4% 1.87% 4.12% 13.68%

gi|9627229|ref|NC_001560.1| 8853 . C T 2.89% 0.52% 7.01% 0% 0.54%

gi|9627229|ref|NC_001560.1| 8920 . A C 0.29% 0.28% 0.82% 0.28% 1.32%

gi|9627229|ref|NC_001560.1| 8922 . G C 0.62% 0.61% 1.53% 0.90% 1.40%

gi|9627229|ref|NC_001560.1| 8925 . G A 0.29% 0.85% 0.88% 0.55% 1.88%

gi|9627229|ref|NC_001560.1| 8971 . C T 1.87% 0.27% 0.54% 0.27% 0.26%

gi|9627229|ref|NC_001560.1| 9337 . AT A TT 4.71% 2.70% 1.13% 2.78% 3.51%

gi|9627229|ref|NC_001560.1| 9342 . T G 0% 0.48% 1.14% 0.57% 0.52%

gi|9627229|ref|NC_001560.1| 9343 . A G 0% 0.27% 1.35% 0.28% 0%

gi|9627229|ref|NC_001560.1| 9414 . G T 0% 1.06% 0.27% 2.24% 0.95%

gi|9627229|ref|NC_001560.1| 9423 . T C 0% 0.26% 0.80% 0% 68.28%

gi|9627229|ref|NC_001560.1| 9523 . C A 0% 0.28% 0% 0.27% 1.31%

gi|9627229|ref|NC_001560.1| 9593 . T C A 0.29% 0.35% 0.28% 0% 18.84%

gi|9627229|ref|NC_001560.1| 9647 . A C 0% 0% 0% 0% 1.70%

gi|9627229|ref|NC_001560.1| 9891 . A G 0% 0.26% 0% 1.82% 0.27%

gi|9627229|ref|NC_001560.1| 9972 . G A 0% 1.82% 0% 0% 0.26%

gi|9627229|ref|NC_001560.1| 10083 . G A 2.36% 0.27% 0.52% 0% 0%

gi|9627229|ref|NC_001560.1| 10149 . A G 3% 0% 0.56% 0% 0%

gi|9627229|ref|NC_001560.1| 10377 . T C 2.86% 0% 0% 0% 0%

gi|9627229|ref|NC_001560.1| 10427 . A AT C 2.55% 1.66% 3.48% 1.11% 1.21%

gi|9627229|ref|NC_001560.1| 10603 . A G 0.27% 13.78% 0.26% 0.26% 0.27%

gi|9627229|ref|NC_001560.1| 11026 . G A 0% 0% 0% 2.14% 0%

gi|9627229|ref|NC_001560.1| 11135 . T A 3.23% 2.33% 2.34% 2.03% 0%

**APPENDIX 6**: Variants present in each of the evolved Random lineages after 10 passages at 40^o^C. CHR: chromosome; POS: position, REF: reference allele; ALT: alternative (variant) allele; VARFREQ: frequency of the variant allele at each lineage.

CHR POS REF ALT VARFREQ

Rnd Rnd Rnd Rnd Rnd

_10A _10B _10C _10D _10E

gi|9627229|ref|NC_001560.1| 412 . C A 0% 0.26% 0% 2.11% 0.79%

gi|9627229|ref|NC_001560.1| 468 . T C 1.15% 0% 0% 0% 0%

gi|9627229|ref|NC_001560.1| 698 . C A 2.11% 0% 0.26% 0% 0%

gi|9627229|ref|NC_001560.1| 966 . G C 0.49% 0.95% 1.19% 1.64% 2.38%

gi|9627229|ref|NC_001560.1| 1016 . A T 10.36% 0% 0% 0.26% 0%

gi|9627229|ref|NC_001560.1| 1066 . G T 0.26% 1.82% 0.26% 0.53% 0.53%

gi|9627229|ref|NC_001560.1| 1228 . C G 0.58% 2% 1.06% 0% 1.07%

gi|9627229|ref|NC_001560.1| 1338 . T G 0.27% 0% 0.27% 4.28% 0.82%

gi|9627229|ref|NC_001560.1| 1395 . C A 8.99% 48.50% 0.54% 0% 19.89%

gi|9627229|ref|NC_001560.1| 1459 . G A 0% 6.96% 0% 0% 0.26%

gi|9627229|ref|NC_001560.1| 1525 . G T 1.89% 0.53% 1.32% 0.52% 0%

gi|9627229|ref|NC_001560.1| 1594 . G T 0.86% 3.37% 1.40% 0.42% 0.88%

gi|9627229|ref|NC_001560.1| 1653 . C T 0.41% 0.82% 0.31% 1.03% 3.94%

gi|9627229|ref|NC_001560.1| 1800 . A C 0.52% 1.06% 0.97% 0.31% 0.31%

gi|9627229|ref|NC_001560.1| 1802 . C G 0.54% 2.08% 0.88% 0.30% 0.28%

gi|9627229|ref|NC_001560.1| 1803 . C T 0.47% 1.24% 0.53% 0.26% 0.26%

gi|9627229|ref|NC_001560.1| 1806 . G A 0% 2.25% 0.84% 0.28% 0.27%

gi|9627229|ref|NC_001560.1| 1877 . C A 0.45% 0% 0.26% 1.58% 1.03%

gi|9627229|ref|NC_001560.1| 1879 . T G 0% 0% 0% 0% 3.86%

gi|9627229|ref|NC_001560.1| 2533 . A G 0.52% 0.83% 1.07% 1.34% 0.80%

gi|9627229|ref|NC_001560.1| 2943 . A G 0.26% 0.26% 0% 4.15% 0.77%

gi|9627229|ref|NC_001560.1| 2971 . C T 0.53% 0.27% 0% 0.27% 5.95%

gi|9627229|ref|NC_001560.1| 3085 . G T 0.27% 0.26% 0% 0.28% 0%

gi|9627229|ref|NC_001560.1| 3098 . A G 16.89% 0% 0% 0% 0%

gi|9627229|ref|NC_001560.1| 3099 . GC G TC 3.39% 2.54% 4.34% 4.87% 2.58%

gi|9627229|ref|NC_001560.1| 3114 . G C 3.11% 2.10% 1.61% 1.57% 1.45%

gi|9627229|ref|NC_001560.1| 3148 . A G 0.26% 0.52% 2.56% 15.60% 28.39%

gi|9627229|ref|NC_001560.1| 3268 . G T 0% 0.26% 1.06% 5.79% 13.91%

gi|9627229|ref|NC_001560.1| 3440 . G C 0.26% 0% 0.53% 0.27% 2.31%

gi|9627229|ref|NC_001560.1| 3445 . C G 0.85% 1.12% 0.29% 2.05% 0.83%

gi|9627229|ref|NC_001560.1| 3451 . T C 0.94% 1.50% 0.95% 2.80% 1.57%

gi|9627229|ref|NC_001560.1| 3452 . C T 1.07% 1.32% 0.53% 1.87% 1.05%

gi|9627229|ref|NC_001560.1| 3453 . C T 0.77% 1.80% 1.04% 2.36% 2.04%

gi|9627229|ref|NC_001560.1| 3456 . C G 0.78% 1.30% 0.78% 2.11% 1.81%

gi|9627229|ref|NC_001560.1| 3459 . C G 0.52% 1.30% 0.79% 1.06% 0.26%

gi|9627229|ref|NC_001560.1| 3461 . A C 1.27% 1.54% 2.06% 2.08% 0.77%

gi|9627229|ref|NC_001560.1| 3462 . A G 0.32% 2.22% 0.66% 1.70% 0.32%

gi|9627229|ref|NC_001560.1| 3463 . G GCTC A 0.78% 2.12% 1.06% 1.32% 0.51%

gi|9627229|ref|NC_001560.1| 3464 . T A G 0.77% 1.83% 0.52% 1.83% 1.30%

gi|9627229|ref|NC_001560.1| 3465 . T C 0.56% 1.93% 0.57% 1.97% 1.12%

gi|9627229|ref|NC_001560.1| 3466 . G C A 1.31% 2.11% 1.31% 2.09% 1.59%

gi|9627229|ref|NC_001560.1| 3467 . T A 0.52% 1.84% 1.30% 2.60% 1.57%

gi|9627229|ref|NC_001560.1| 3472 . A G 0.27% 0.26% 0.26% 1.07% 0.78%

gi|9627229|ref|NC_001560.1| 4074 . A C 1.37% 1.64% 0.83% 1.11% 1.10%

gi|9627229|ref|NC_001560.1| 4148 . A C 6.08% 44% 91.47% 37.67% 14.63%

gi|9627229|ref|NC_001560.1| 4180 . C A 99.23% 98.73% 98.45% 75.98% 68.62%

gi|9627229|ref|NC_001560.1| 4330 . C A 1.04% 0.77% 0% 0% 0.51%

gi|9627229|ref|NC_001560.1| 4390 . G C 97.83% 97.33% 96.45% 82.91% 77.98%

gi|9627229|ref|NC_001560.1| 4395 . T A 0.42% 0.85% 0.79% 1.03% 1.01%

gi|9627229|ref|NC_001560.1| 4413 . C A 1.06% 0.26% 0% 0.26% 0.27%

gi|9627229|ref|NC_001560.1| 4488 . A C 9.67% 53.48% 0.54% 0.54% 17.21%

gi|9627229|ref|NC_001560.1| 4694 . T A 1.14% 0.28% 1.77% 0% 0%

gi|9627229|ref|NC_001560.1| 4729 . A T 0.28% 0% 1.93% 3.05% 0.27%

gi|9627229|ref|NC_001560.1| 4924 . G A 0.55% 2.41% 0.27% 0% 0%

gi|9627229|ref|NC_001560.1| 4978 . A T 0.27% 0% 0.27% 0% 8.87%

gi|9627229|ref|NC_001560.1| 5345 . C G 2.08% 0.85% 0.29% 0.80% 1.34%

gi|9627229|ref|NC_001560.1| 5347 . C T 2.43% 1.24% 0% 1.97% 1.90%

gi|9627229|ref|NC_001560.1| 5425 . G A 15.40% 0.26% 0.77% 0.26% 0.51%

gi|9627229|ref|NC_001560.1| 6532 . A C 0% 0.26% 4.71% 3.64% 0%

gi|9627229|ref|NC_001560.1| 6681 . A C 0.27% 1.94% 0% 0% 0%

gi|9627229|ref|NC_001560.1| 6844 . C T 0.80% 1.34% 0% 0.81% 1.61%

gi|9627229|ref|NC_001560.1| 6859 . C T 0.77% 0.26% 0.51% 1.31% 0.77%

gi|9627229|ref|NC_001560.1| 6895 . G A 0% 0% 1.57% 9.16% 20%

gi|9627229|ref|NC_001560.1| 7077 . T A 0% 0.37% 0.43% 1.40% 0.43%

gi|9627229|ref|NC_001560.1| 7078 . C T 0.29% 1.10% 0.28% 0.56% 0.82%

gi|9627229|ref|NC_001560.1| 7079 . C T 0% 0.54% 0.28% 1.46% 0.57%

gi|9627229|ref|NC_001560.1| 7085 . G A 0% 0.27% 0.27% 1.06% 0.54%

gi|9627229|ref|NC_001560.1| 7105 . C T 2.29% 0.29% 0.58% 0.31% 0%

gi|9627229|ref|NC_001560.1| 7390 . A C 0% 0.81% 0.26% 1.02% 0%

gi|9627229|ref|NC_001560.1| 7429 . C A 1.41% 0.56% 0.57% 0.62% 0.30%

gi|9627229|ref|NC_001560.1| 7536 . T G 1.83% 0% 0% 0.54% 0.53%

gi|9627229|ref|NC_001560.1| 7570 . A G 9.70% 50% 0.26% 0.27% 14.36%

gi|9627229|ref|NC_001560.1| 7698 . C T 0.54% 0.84% 0.53% 0.27% 1.88%

gi|9627229|ref|NC_001560.1| 7701 . T A 0.52% 0.80% 0.52% 0.27% 1.87%

gi|9627229|ref|NC_001560.1| 7708 . G C 0.52% 0.53% 0.26% 0.27% 1.82%

gi|9627229|ref|NC_001560.1| 7912 . A C 0.28% 0.27% 0.27% 0.26% 9.23%

gi|9627229|ref|NC_001560.1| 8371 . C T 1.10% 0.28% 0.84% 0% 0.55%

gi|9627229|ref|NC_001560.1| 8624 . G A 1.16% 0% 0.54% 1.09% 0.54%

gi|9627229|ref|NC_001560.1| 8749 . G C 13.68% 10.85% 8.73% 1.83% 5.03%

gi|9627229|ref|NC_001560.1| 8852 . G A 0.28% 0.54% 0.80% 3.99% 1.37%

gi|9627229|ref|NC_001560.1| 8853 . C T 0.54% 0.27% 0.26% 12.53% 0.80%

gi|9627229|ref|NC_001560.1| 8920 . A C 1.32% 0.54% 0.27% 0.55% 0.28%

gi|9627229|ref|NC_001560.1| 8922 . G C 1.40% 0.57% 0.29% 1.53% 1.55%

gi|9627229|ref|NC_001560.1| 8925 . G A 1.88% 0.54% 0.27% 0.84% 1.45%

gi|9627229|ref|NC_001560.1| 8971 . C T 0.26% 0% 1.06% 2.42% 0.27%

gi|9627229|ref|NC_001560.1| 9320 . CA C TA 0.32% 1.08% 0.26% 2.90% 0.54%

gi|9627229|ref|NC_001560.1| 9321 . A G 0% 0.57% 0.28% 2.24% 0.29%

gi|9627229|ref|NC_001560.1| 9322 . A G 0% 0.54% 0.78% 1.61% 0.28%

gi|9627229|ref|NC_001560.1| 9325 . C T 0% 1.38% 0.78% 2.86% 0.52%

gi|9627229|ref|NC_001560.1| 9326 . C T 0.33% 1.14% 0.79% 2.94% 0.28%

gi|9627229|ref|NC_001560.1| 9328 . A T 0% 0.85% 0.77% 1.29% 0.80%

gi|9627229|ref|NC_001560.1| 9331 . C T 0% 1.14% 0.77% 3.18% 0.53%

gi|9627229|ref|NC_001560.1| 9332 . A C 0% 1.14% 0.78% 2.17% 0.27%

gi|9627229|ref|NC_001560.1| 9333 . A T 0.33% 0.88% 0.82% 2.25% 0.29%

gi|9627229|ref|NC_001560.1| 9335 . C G 0.97% 1.14% 1.08% 2.19% 0.55%

gi|9627229|ref|NC_001560.1| 9336 . C T 0% 0.91% 0.29% 3.43% 0.60%

gi|9627229|ref|NC_001560.1| 9337 . AT A TT 3.51% 2.04% 2.19% 2.75% 1.42%

gi|9627229|ref|NC_001560.1| 9340 . T G 0% 0.85% 0.80% 2.12% 0.27%

gi|9627229|ref|NC_001560.1| 9342 . T G 0.52% 1% 1.60% 4.21% 0.63%

gi|9627229|ref|NC_001560.1| 9343 . A G 0% 1.16% 0.82% 3.30% 0.28%

gi|9627229|ref|NC_001560.1| 9347 . G T 0% 1.23% 0.86% 3.22% 0.28%

gi|9627229|ref|NC_001560.1| 9348 . G C 0% 1.15% 0.80% 3% 0.26%

gi|9627229|ref|NC_001560.1| 9350 . A T 0% 1.59% 1.10% 4.64% 0.26%

gi|9627229|ref|NC_001560.1| 9352 . A T 0.39% 0% 0.31% 2.26% 0%

gi|9627229|ref|NC_001560.1| 9354 . A C 0% 0.30% 0.27% 1.73% 0%

gi|9627229|ref|NC_001560.1| 9356 . A T 0% 0.30% 0.27% 1.71% 0.26%

gi|9627229|ref|NC_001560.1| 9357 . A C 0% 0.31% 0.28% 1.75% 0%

gi|9627229|ref|NC_001560.1| 9359 . A T 0% 0.31% 0.27% 1.71% 0%

gi|9627229|ref|NC_001560.1| 9414 . G T 0.95% 1.33% 0.93% 1.36% 0%

gi|9627229|ref|NC_001560.1| 9423 . T C 68.28% 0.53% 0.26% 5.70% 2.41%

gi|9627229|ref|NC_001560.1| 9523 . C A 1.31% 0.28% 0.28% 0.28% 0%

gi|9627229|ref|NC_001560.1| 9593 . T C A 18.84% 0.84% 0.29% 0% 13.89%

gi|9627229|ref|NC_001560.1| 9594 . A G 79.94% 98.15% 98.16% 52.65% 40.06%

gi|9627229|ref|NC_001560.1| 9647 . A C 1.70% 0.80% 0% 0.53% 0%

gi|9627229|ref|NC_001560.1| 9972 . G A 0.26% 0% 0% 0% 1.05%

gi|9627229|ref|NC_001560.1| 10083 . G A 0% 0.27% 1.08% 4.71% 0.27%

gi|9627229|ref|NC_001560.1| 10149 . A G 0% 0% 5.06% 7.73% 0%

gi|9627229|ref|NC_001560.1| 10427 . A AT C 1.21% 1.10% 1.42% 2.52% 3.72%

gi|9627229|ref|NC_001560.1| 10603 . A G 0.27% 1.06% 0.27% 0.54% 2.95%

gi|9627229|ref|NC_001560.1| 10767 . A G 0% 0.27% 0% 0% 6.91%

gi|9627229|ref|NC_001560.1| 10892 . G A 0% 0% 0.26% 0.26% 6.54%

gi|9627229|ref|NC_001560.1| 10893 . A G 0% 0.26% 0.26% 0.53% 2.11%

gi|9627229|ref|NC_001560.1| 11135 . T A 0% 1.32% 3.36% 3.55% 1.96%
